# Interventions to minimize periodontal defect distal to second molar after mandibular third molar surgery: an overview of systematic reviews

**DOI:** 10.1007/s10006-025-01432-5

**Published:** 2025-08-22

**Authors:** Ioulianos Apessos, Christodoulos Dovas, Spyridon Mantalenakis, Theodoros Lillis, Georgios Antonoglou

**Affiliations:** 1 Department of Dentoalveolar Surgery, Implantology and Oral Radiology, School of Dentistry, Faculty of Health Sciences, Aristotle University of Thessaloniki, Thessaloniki, 54124 Greece; 2 Division of Dentistry, 424 General Military Training Hospital, Thessaloniki, Greece; 3 Periodontology Unit, Centre for Host Microbiome Interactions, Faculty of Dentistry, Oral and Craniofacial Sciences, King’s College London, London, UK

**Keywords:** Oral surgical procedures, Periodontal diseases, Molar, third/surgery, Systematic reviews (as topic)

## Abstract

**Purpose:**

This overview summarized different interventions that were performed for minimizing periodontal defects distal to the mandibular second molar and improve hard and soft tissue healing after third molar surgery.

**Methods:**

Literature search was conducted in the following 9 databases: Medline (via Pubmed), ScienceDirect, Scopus, Virtual Health Library, Wiley Online Library, Web of Science, ProQuest Dissertations and Theses Global and Google Scholar. Systematic reviews with or without meta-analysis investigating the effect of different interventions on soft and hard tissue healing after third molar surgery were considered. Methodological quality of included reviews was assessed with AMSTAR-2 tool. The degree of overlapping of index publications in the eligible reviews was presented and calculated with the GROOVE tool.

**Results:**

Thirty-three reviews were included, collectively encompassing 191 distinct primary studies that evaluated flap design, extraction socket management, and postsurgical care. Quantitative data retrieved from the methodologically rigorous MAs revealed that the application of autologous platelet concentrates (APCs) is the best choice to improve soft tissue healing [MD = 1.01; 95% CI (0.77, 1.24), 7 days follow-up] and bone mineral density [SMD = 2.34; 95% CI (0.18,4.51), 4 months follow-up] and alleviate pain [SMD= -0.86; 95% CI (-1.26, -0.46), 3 days follow-up], trismus [SMD= -0.26; 95% CI (-0.48, -0.03), 7 days follow-up], alveolar osteitis [RR = 0.43; 95% CI (0.28, 0.65)] and swelling [MD= -1.66; 95% CI (-2.43, -0.90), 3 days follow-up]. Ridge preservation is the most effective intervention in improving pocket probing depth [MD= -1.42; 95% CI (-2.01, -0.83), 6–72 months follow-up], clinical attachment level [MD = 1.98; 95% CI (1.44, 2.52), 4.5-72months follow-up] and alveolar bone height [MD = 1.21; 95% CI (0.21, 2.21), 6–12 months follow-up] distal to mandibular second molar.

**Conclusion:**

Minimizing tissue trauma is key in surgical extractions. Our overview found that triangular flaps reduce PPD, while envelope flaps lower postoperative pain. APCs improved healing and reduced adverse events, and HyA mainly alleviated pain. All regenerative techniques enhanced periodontal outcomes, though high heterogeneity and variable study quality urge cautious interpretation.

**Supplementary Information:**

The online version contains supplementary material available at 10.1007/s10006-025-01432-5.

## Introduction

Surgical extraction of mandibular third molar (M3M) is one of the most common oral surgical procedures. Recurrent pericoronitis, non-restorable dental caries, infection, odontogenic cyst, localized periodontitis distal to mandibular second molar (M2M), pathological dental migration/movement, occlusal alteration, malposition and even prophylactic reasons are numbered among indications of its surgical extraction [1].

Optimal management of M3Ms’ extraction site continues to challenge dentists. An important issue to be addressed is the post-extraction defect and soft tissue collapse that compromise periodontal health of M2M. Additionally, surgical trauma leads to pain, facial swelling and trismus, while complications like alveolar osteitis, infection and fracture of the mandible may occur postoperatively [2].

Several studies have documented risk factors for periodontal health distal to M2M that are related to the presence and surgical extraction of M3M. Angulation and impaction depth of M3M, age, smoking, operator’s experience, preoperative periodontal condition and oral hygiene are among them. Semi-impacted M3Ms are more prone to periodontal infection and greater attachment loss whereas deeply impacted M3Ms lead to periodontal defects after their surgical removal [3].

Subsequently, in the last 50 years different techniques and biomaterials have been suggested for the prevention and treatment of intraosseous defects, deep periodontal pockets and adverse events. Different flap designs, guided bone regeneration (GBR), guided tissue regeneration (GTR) and autologous platelet concentrates (APCs) are among them. One of the simplest and most common techniques to enhance healing is the use of collagen sponges, some of which are embedded with topical solutions of medications such as antibiotics [4]. These sponges aid in clot stabilization, support tissue regeneration, and help reduce postoperative complications, making them a widely adopted adjunct in clinical practice [5]. The previous led to a plethora of systematic reviews of different interventions for the same clinical scenario.

Thus, the aim of the present overview was to summarize the effectiveness of different interventions in order to minimize the periodontal defect distal to M2M and improve hard and soft tissue healing after surgical extraction of M3M.

## Methods

### Protocol and registration

The protocol of this overview of reviews was registered in the International Prospective Register of Systematic Reviews (PROSPERO) database (registration ID; https://www.crd.york.ac.uk/PROSPERO/view/CRD42022307614). Reporting followed Preferred Reporting Items for Overviews of Reviews (PRIOR) guidelines [6]. 

### Information sources and search strategy

The following databases were searched, using a combination of keywords: Medline (via Pubmed), ScienceDirect, Scopus, Virtual Health Library, Wiley Online Library, Web of Science, ProQuest Dissertations and Theses Global and Google Scholar. The last search date was August 15, 2024. No language restriction was used. The original search strategy was created for PubMed and adjusted thereafter to the other databases (Table S1, Supplementary file 1).

### Eligibility criteria

#### Population

Adolescent or adult patients with impacted mandibular third molars of any depth and orientation, with any diagnosis indicative for surgical extraction were included.

#### Interventions

We included studies investigating operative and postoperative interventions designed to improve periodontal status distal to M2M following the surgical extraction of M3M. To enhance clarity, interventions were categorized into three distinct groups.

The first category addressed surgical access and closure methods, including variations in flap design and soft tissue suturing techniques, as well as the selection of suture materials to facilitate proper wound healing and minimize postoperative complications.

The second category focused on techniques, encompassing factors such as the type of local anesthesia administered and extraction socket management utilizing APCs and various biomaterials to optimize healing outcomes.

Finally, post-surgical maneuvers involved oral hygiene protocols, systemic antibiotic prescriptions, the timing of suture removal, and mechanical biofilm and calculus removal from the distal aspect of M2M to support periodontal stability and promote optimal recovery.

#### Comparators

Spontaneous healing, placebo and comparison of different interventions with each other were included.

#### Outcomes

Primary outcomes included soft and hard tissue healing. Outcome measures included any soft or hard tissue healing index, change in probing pocket depth (PPD), clinical attachment level (CAL) and alveolar bone level (ABL) from baseline (before extraction) to the last available follow-up (after extraction). Secondary outcomes included adverse events, including pain, trismus, alveolar osteitis (AO) and swelling.

#### Study design

The unit of analysis of this overview were systematic reviews (SRs) with or without meta-analysis (MA) of randomized controlled trials (RCTs) or non-randomized studies of interventions (NRSIs).

#### Study selection and data management

Literature search results were uploaded by one author (IA) to Distiller Systematic Review (DSR) software, an Internet based software program that facilitates collaboration among authors during the study selection process. After duplicates’ removal, two authors (IA, CD) screened the titles and/or abstracts of studies retrieved using the search strategy and those from additional sources (hand searching) to identify articles that potentially met the inclusion criteria. The full text of these potentially eligible studies, as well as of those abstracts which do not provide sufficient information to allow decision-making as regards inclusion or exclusion, were retrieved and independently assessed. Any disagreement was resolved by discussion with a third reviewer (GA). The same two independent authors (IA, CD) collected data from eligible SRs and their primary studies. The following data were extracted and recorded: Basic information about SRs (title, authors, year of publication, number of studies and participants included), basic information about primary studies (authors, year of publication, study design, country of publication), SRs’ search strategies (number and names of databases searched, date ranges of databases searched, date of last search update), SRs’ population(s) (participants’ characteristics such as age, sex, setting), SRs’ interventions (type of intervention, dose, frequency, duration), SRs’ comparators (type of comparator, dose, frequency, duration), primary and secondary outcomes (as specified in Methods section of the SRs), additional information (author’s comments, SR limitations, and methodological quality/risk of bias).

Overlap of primary studies among SRs included in our overview was assessed with the GROOVE (Graphical Representation of Overlap for OVErviews) tool [7]. Starting from a matrix of evidence, GROOVE provides the number of included primary studies and SRs included in the matrix; the absolute number of overlapped and non-overlapped primary studies; and an overall corrected covered area (CCA) assessment [8, 9]. The tool also provides a detailed CCA assessment for each possible pair of SRs, with a graphical representation of these results. The formula for calculating CCA is: CCA = (N − r)/(rc − r), where N = number of total primary studies, including double counting, r = number of unique studies (number of rows), c = number of included SRs (number of columns). CCA of 0–5% represents a slight overlap, 6–10% a moderate overlap, 11–15% a high overlap, and above 15% a very high overlap [9]. Should high or very high overlap be detected, which is interpreted as CCA equal to or more than 10%, we planned to retain the review which is (1) the most recent, (2) containing a higher amount of information, and (3) the most rigorous in terms of methodology, as assessed by AMSTAR 2 tool.

In order to avoid skewed reporting, we presented in tables quantitative data of the most comprehensive reviews with high methodological quality. As adverse events were our secondary outcome, we extracted relative data only from reviews that assessed as an outcome both parameters of healing and adverse events, so the existence of extra studies that specify their topic to adverse events’ avoidance cannot be excluded.

#### Methodological quality and risk of bias assessment

The methodological quality of included SRs was assessed with the AMSTAR 2 tool (A MeaSurement Tool to Assess systematic Reviews) [10]. The assessment was performed by two reviewers (IA, CD) and any discrepancies were resolved by a third reviewer (SM). AMSTAR2 includes the following critical domains: protocol registered before start of review; adequacy of literature search; justification for excluded studies; risk of bias for included studies; appropriateness of meta-analytic methods; consideration of risk of bias when interpreting results; and assessing presence and likely impact of publication bias. The tool provides guidance to rate the overall confidence in the results of a review (high, moderate, low or critically low depending on the number of critical flaws and/or non-critical weaknesses). Data on risk of bias of primary studies contained within included SRs were extracted and tabular summaries of the assessments were provided.

#### Data synthesis and analysis

Quantitative outcome data (including mean difference, effect estimates and 95% confidence intervals) regarding wound healing indices, PPD, CAL and ABL and adverse events contained within each included SR are presented with narrative summaries and corresponding tables. If no MA was performed (systematic reviews with a narrative synthesis) for the outcome of interest, tabular summaries of qualitative findings were provided.

#### Reporting bias and certainty of evidence assessment

Data on publication bias detection and overall quality of evidence included in SRs and judged with any validated tool were extracted and tabular summaries of the assessments were provided. If we identified discrepant data across systematic reviews, we planned to extract data from the most methodologically rigorous review.

## Results

### Study selection and characteristics

**Fig. 1:**
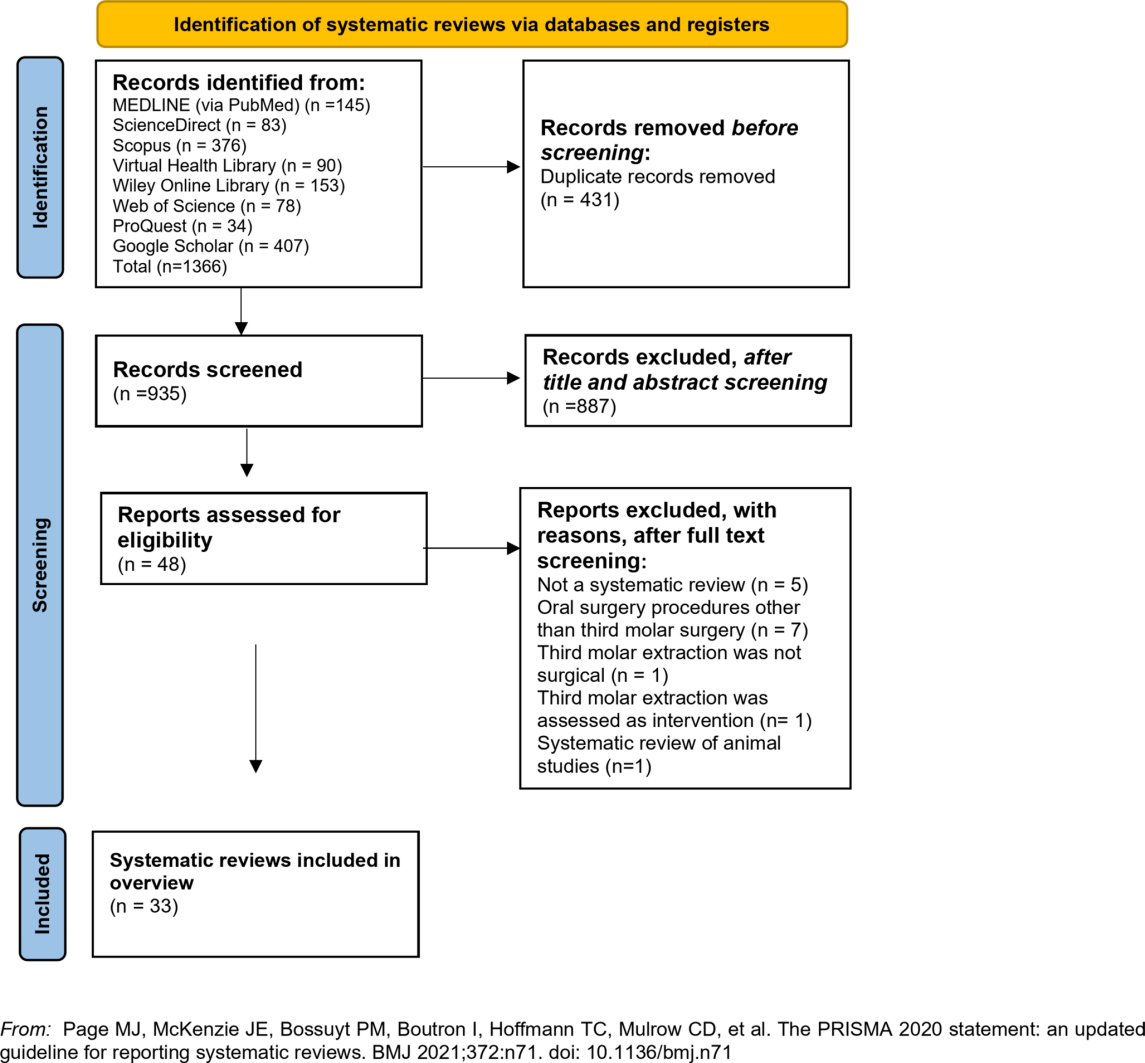
Simplified PRIOR flow diagram

Literature search yielded 1366 records. After duplicates’ removal and title/abstract screening, 48 records retrieved in full text. Fifteen studies were subsequently excluded with reasons [11–25], leaving 33 [3, 26–57] reviews to be included in this overview (Fig. 1*)(Table **S2*). Table 1 summarizes the main characteristics of the included reviews. Twenty-two were SRs with MA [3, 26–29, 31, 33, 34, 36–39, 43, 45, 47, 48, 51, 52, 54, 55, 57], nine were SRs with narrative synthesis [30, 32, 40–42, 49, 50, 53, 56] and two were SRs with network MA [35, 46]. These were published from 2010 to 2024. Authors’ affiliations were located at China [26, 28, 44, 45, 54, 55, 57], Brazil [31, 36, 46, 51, 53], Spain [3, 30, 32, 33], Italy [35, 39, 42, 47], USA [34, 40], Denmark [48, 49], Egypt [29], Hong Kong [37], Taiwan [38], Portugal [41], Japan [43], United Kingdom [50], Austria [52] and France [56].

**Table 1:** Characteristics of the included studies

Author, year, country, study design	Number and type of included primary studies number of participants/sites (Intervention and Control), country and publication year range	Databases, date of last search or search date range, search limitations	Age, sex, setting	Systematic review’s interventions vs. systematic review’s comparators	Primary and secondary outcomes	Certainty of evidence, reporting biases
Xiang et al. 2019, China, systematic review and meta-analysis	10 RCTs (385 and 597) 4 in Turkey 1 in Iran 2 in India 2 in Cyprus 1 in Egypt published from 2010 to 2017	PubMed, Embase, Cochrane library Limited to English language, last search on September 3, 2017	Mean age 24.12 m/f 173/212 outpatients University Dental Clinic	PRF vs. Spontaneous healing	Adverse events (pain, alveolar osteitis, trismus), osteoblastic activity, soft tissue healing	Quality of the evidence (GRADE) moderate for swelling outcome, low for pain, alveolar osteitis and soft tissue healing and very low for trismus and osteoblastic activity. No evidence of asymmetrical distribution in the funnel plot in trismus (Begg’s test *P* = 0.734; Egger’s test *P* = 0.677) and alveolar osteitis (Begg’s test *P* = 1.000; Egger’s test *P* = 0.198)
Chen et al. 2016, USA, systematic review and meta-analysis	8 RCTs [224 (35 dropped out) and 383(70 dropped out)] 2 in Italy 2 in Turkey 1 in Iran 1 in Jordan 1 in Germany 1 in Brazil published from 2003 to 2015	MEDLINE (PubMed), Embase, Cochrane CENTRAL, Scopus, oral surgery related international journals and reference list of selected articles. Search until April 2016	Age 15–41 (20.73) 7 University Dental Clinic and 1 University Hospital	Flaps other than triangular. Szmyd flap in 1 study Szmyd modified in 2 studies Envelope in 3 studies Envelope modified in 1 study Triangular modified in 1 study Vs Triangular flap	Change in PD and CAL	There was no significant publication bias detected for WDPDR (Egger’s test, *P* = 0.80). The funnel plot demonstrated a symmetric distribution of the data in each study
He et al., China, 2017, systematic review and meta-analysis	9 RCTs and 1 NRSI (retrospective case control) (483 and 935) 3 in Turkey 2 in Cyprus 2 in India 1 in Iran 1 in USA 1 in Italy published from 2010 to 2016	PubMed, Web of Science, Embase, Cochrane Library. Search until October 2016	Age range (mean) 17–50 (25.77). Age range NM in 2 studies and mean in 3. m/f 213/248, sex NM in 2 studies 6 University Dental Clinic, 1 Hospital Dental Department, 2 University Hospital, 1 private practice	PRF vs. Spontaneous healing	Adverse events (pain, swelling, trismus, alveolar osteitis) and osteoblastic activity	The funnel plot showed that considerable publication bias only existed in the comparisons of pain
Al-Hamed et al., Egypt, 2017, systematic review and meta-analysis	5 RCTs and 1 NRSI (183 and 343) 2 in India 2 in Turkey 1 in Iran 1 in Cyprus published from 2010 to 2015	PubMed, the Cochrane Central Register of Controlled Trials, and Scopus. Search until November 2015	Age range (mean) 18–50 (25.75). Age range NM in 1 study 6 University Dental Clinic	PRF vs. Spontaneous healing	PPD, Bone healing, soft tissue healing, adverse events (pain, swelling, trismus, alveolar osteitis)	NR
Barona- Dorado et al., Spain, 2014, systematic review	3 RCTs (58 and 116) 1 in Turkey 1 in USA 1 in Iran published from 2008 to 2012	Medline (via Pubmed), EMBASE (via Ovid), NIH, and the Cochrane Central Register of Controlled Trials Search until 30 June 2013	Age range 18–45, NM in 1 study Mean age 21.8, NM in 2 studies 2 in University Dental Clinic 1 in private practice	PRP vs. Spontaneous healing	Adverse events (pain, alveolar osteitis), tissue healing, osteoblastic activity	Jadad Scale (Blind assessment of the quality of trial reports) for quality evaluation. Two studies scored 3 and one 4. Quality of evidence poor for all outcomes.
Lopes da Silva et al., Brazil, 2020, systematic review and meta-analysis	20 RCTs (713 and 1265) 3 in Iran 1 in Saudi Arabi 1 in Jordan 3 in India 5 in Turkey 2 in Brazil 1 in Austria 2 in Italy 1 in New Zealand 1 in USA published from 2002 to 2018	Web of Science, PubMed (MEDLINE), Virtual Health Library (VHL; LILACS and IBECS), Cochrane Library, and Scopus. Search until November 2018	Age range 15–61, NM in 1 study Mean age 23.3, NM in 5 studies m/f 249/464, NM in 3 studies 19 in University Dental clinic, setting NR in one study	Triangular flap vs. Envelope flap	Primary: Pain, oedema, trismus Secondary: wound dehiscence, ecchymosis, alveolar osteitis, periodontal condition, and duration of surgery	NR
Aloy- Prosper et al., Spain, 2010, a systematic review	8 RCTs (180 and 269) 2 in Spain 1 in China 1 in Brazil 1 in Sweden 1 in USA 1 in Italy 1 in Turkey published from 2000 until 2009	PubMed and oral surgery related international journal from Jan 1997 until March 2009	Age range 16–52, NM in 2 studies Mean Age 28.18, NM in one study m/f 52/80, NM in 3 studies 8 in University dental Clinic	Dental Hygiene distal to M2M	PPD, CAL	NR
				-scaling with hand instruments, mouth rinse and oral hygiene control -scaling with ultrasound device vs. no scaling		
				Flap design		
				-Bayonet flap -Bayonet flap to 2 mm -Bayonet flap Vs -Szmyd flap -Bayonet flap -Szmyd modified flap		
				Bone regeneration techniques		
				- Resorbable membrane - Resorbable membrane or Demineralized bone powder -PRP Vs -Non Resorbable membrane -Spontaneous healing -Spontaneous healing		
Toledano-Serrabona et al., Spain, 2021, a systematic review and meta-analysis	3 RCTs (81 and 162) 1 in Brazil 1 in Italy 1 in Saudi Arabia published from 2009 until 2012	PubMed, Scopus and Cochrane Library Search until April 2020 Oral surgery related journals from 2010 to 2020	Age range 15–35 Mean age 32, NM in 2 studies m/f 43/38 3 in University dental clinic	Xenograft and collagen membrane or xenograft alone vs. spontaneous healing	PPD reduction, CAL gain, ABL gain, adverse events	
Lee et al., USA, 2016, a systematic review and meta-analysis	7 RCTs (152p and 284 s) 3 in Italy 2 in USA 1 in Saudi Arabia 1 in Iran published from 1993 until 2014	MEDLINE (PubMed), Embase, Web of Science, Dental and Oral Sciences Source, oral surgery related journals from January 1960 to August 2015, as long as reference lists of selected articles	Age range 21–35, NM in one study Mean age 28.82, NM in 2 studies m/f 49/59, NM in 2 studies 6 in University Dental Clinic 1 in private practice	Bone grafting, or GTR, or combination technique (bone grafting plus membrane) Vs spontaneous healing or other regenerative technique	CAL gain, PPD reduction	Evidence level 2b for 6 studies and 1b for 1 study based on the Oxford Centre for Evidence-based Medicine. Egger test showed no significance in regards to WDCAL and WDPD. Funnel plots did not demonstrate symmetric distribution of the data, meaning publication bias could not be completely ruled out.
Barbato et al., Italy, 2016, a systematic review and network meta-analysis	16 RCTs (317p and 509 s) 5 in Italy 4 in USA 2 in Canada 1 in Iran 1 in Turkey 1 in China 1 in Spain 1 in Saudi Arabia published from 1985 until 2013	PubMed MEDLINE, Cochrane Central Register of Controlled Trials (CENTRAL), and EMBASE (via OVID), from 1974 to 22 December 2014	Age range 16–59, NM in 4 studies Mean age 27.84, NM in 5 studies m/f 65/106, NM in 7 studies 13 in University Dental Clinic 1 in Hospital Dental Departent 1 in Hospital Dental Department and in University Hospital Dental Clinic 1 in private dental practice	regenerative/grafting procedures flap design type of suturing periodontal care of M2M vs. spontaneous healing or different regenerative technique, other flap design, other suturing technique, no periodontal maintenance	CAL gain, PD reduction	Overall quality of evidence was rated from very low to moderate.
Camps-Font et al., Spain, 2018, a systematic review and meta-analysis	21 RCTs [621p (42 dropped out) and 795 s] published 6 in Italy 4 in USA 2 in Brazil 2 in Iran 2 in India 1 in Spain 1 in China 1 in Egypt 1 in Sweden 1 in Turkey pubished from 1993 until 2017	MEDLINE (via PubMed), Cochrane Library (Wiley), Scopus (Elsevier), and Web of Science (Thomson Reuters), ClinicalTrials.gov,41 OpenGrey,42 and the World Health Organization’s International Clinical Trial Registry Platform Last search on 1 September 2017	Mean age 28.16, NM in 8 studies Age range 15–55, NM in	Platelet concentrates, GTR, Osseous grafting and GBR Vs Other periodontal regenerative procedures and spontaneous healing	CAL gain, PD reduction, ABL gain and adverse event rate	The Peters test showed no significance (t = 1.55, *P* = 0.219), visual inspection of the funnel plot showed a slight asymmetry of data. The possibility of publication bias could not be ruled out.
Ramos et al., Brazil, 2022, a systematic review and meta-analysis	17 RCTs (510p and 705 s) 6 in India 5 in Turkey 1 in Germany 1 in Italy 1 in Brazil 1 in Egypt 1 in Cyprus 1 in Lithuania published from 2015 until 2021	PubMed, MEDLINE, EMBASE, Web of Science, Virtual health library (BVS), and Cochrane, search until 30 July 2021	Age range 17–40 Mean age 23.96 m/f 143/180, NM in 6 studies	PRF, A-PRF, L-PRF Vs spontaneous healing	pain, edema, trismus, soft tissue healing, periodontal regeneration	NR
Pang et al., Hong Kong, 2022, a systematic review and meta-analysis	8 RCTs 6 Prospective Analysis 859(73) patients 413(14) test sites/383(20) control sites 4 studies with no data on test/control sites. 4 in Italy 3 in Spain 1 in Germany 1 in Iran 1 in Chaina 1 in Hong Kong 1 in Taiwan 1 in Vietnam 1 in India Published from 2004–2020	Cochrane Central Register of Controlled Trials (CEN TRAL), MEDLINE (through PubMed), Scopus (Elsevier), and Embase. Search until April 9 2020, manual search in “Periodontology 2000, “Journal of Clinical Periodontology,” “Journal of Periodontology,” “International Journal of Oral and Maxillofacial Surgery,” “Journal of Oral and Maxillofacial Surgery,” for relevant articles published from April 2011 to March 2020. No mentioned search limitations.	Age range: 15–81 Mean age in studies: 15.9–37.2 m/f 331/387 NM in 4 studies 13 University setting 1 Hospital	−3-sided flap -SRP at the distal of second molar -regenerative materials -Postoperative use of antibiotics antiseptic mouth rinse vs. -envelope or 2-sided -without periodontal treatment -spontaneous healing -without the use of antibiotics -without antiseptic mouth rinse	Final PPD, CAL changes, Final CAL, ABD (alveolar bone defect) reduction, Final ABD, Baseline PDD	NR
Soo-Hoong Low et al., 2020 Taiwan, systematic review and meta-analysis	18 RCTs 9 parallel 9 split-mouth 475p (537 test sites and 237 control) 4 in Italy 3 in Spain 2 in USA 2 in Iran 2 in Turkey 1 in China 1 in Saudi Arabia 1 in Sweden 1 in Germany 1 in India Published from 1993 to 2020	Medline, Embase, Scopus, and Google Scholar, last search was conducted in Oct. 2019.	Age range: 15–55 Mean age range: 21.03-43 129 males 151 females 7 articles don’t mention All University setting	e-PTFE membrane resorbable PLA barrier demineralized bone powder ultrasonic root debridement polyglycolic acid/polylactic acid bioresorbable membranes 3-cornered flap Szmyd flap (anchor suture flap) Envelope flap Bovine porous bone mineral (BPBM) alone, BPBM plus collagen membrane (CM) xenograft plus a membrane Lincomycin-Impregnated Demineralized Freeze-Dried Bone Allograft Surgical extraction Vs distal root surface debrided triangular flap spontaneous healing	Ridge preservation in sites where initial PD < 5 mm, 5–7 mm and > 7 mm (reduction of second molar distal site PD)	NR
Franchini M et al., 2019, Italy, systematic review and meta-analysis	3 RCTs (202, 188) 1 in India 1 in Spain 1 in Nigeria published from 2012 to 2015	PubMed, Embase, Scopus, Ovid, Cochrane library Original, Rcts with adults patients, English language in the last 20 years Last search 30 May 2019	Mean age 27,4 (18–50) m/f 91/111 n mention in. s	PRP vs. Spontaneous healing	Probing Depth, Clinical Attacment Level, Gingival Recession, Bone Defect	NR
Miron RJ et al.,2016 USA, systematic review	1 CCT(100,200) 1 RCT(20,40)	MEDLINE Last search 7th April 2016	Mean age n/m m/f n/m Setting n/m	PRF vs. PRP Spontaneous Healing	Soft Tissue Healing Localized Osteitis	NR
Danylyuk Y., Portugal 2019, systematic review	24 RCTs (497/993) 6 Turkey 1 Iran 2 Cyprus 2 India 1 Egypt 1 Lithuania 1 Brazil	MEDLINE (Pubmed) Google Scholar May 2019? (Last article included) Limitations: only RCTs using L-PRF	M/F impossible to analyze as many studies don’t provide information. Mean age: 23.3 (15–48) outpatients	L-PRF vs. Spontaneous healing	Alveolitis Post-op pain Oedema Osseous regeneration Probing depth Trismus Healing of soft tissues(Healing index score)	NR
Del Fabbro M et al. 2011, Italy, systematic review	3 CCTs (34,72) 2 in USA 1 in India 3 RCTs (48,96) 2 in Turkey 1 in Italy published from 2005 to 2010	MEDLINE, EMBASE, Cochrane Central Register of Controlled Trials (A further hand search was performed on the main international journals in the field of dentistry and of oral and maxillofacial surgery Radiology and Endodontology). No language or time restriction No limitation about follow up and number of patients Last search October 2010	Mean Age 24,05(No mention in 2 studies) (18–45) (No mention in 2 studies) m/f not reported Setting not reported	PRP PRGF PRP + Gelfoam Vs Spontaneous Healing Gelfoam	Soft tissue healing Hard tissue healing Post-op quality of life Tissue markers of wound healing and inflammation	NR
Fujioka-Kobayashi M et al. 2021 Japan, systematic review and meta-analysis	18 RCTs (643,1097) 7 in Turkey 4 in India 2 in Cyprus 1 in Italy 1 in Iran 1 in Lithuania 1 in Brazil 1 in Saudi Arabia Published from 2010 to 2019	PubMed/MEDLINE, the Cochrane Central Register of Controlled Trials, Scopus, Embase and Lilacs Grey Literature (Literature Report and Opengrey) Hand search Last June 2020 without other restrictions regarding date or language.	Mean Age 23,63(No mention in 4 studies) m/f 199/323 (No mention in 3 studies)	PRF Piezo Piezo + PRF PRF + HA vs. Clot Traditional Piezo + Clot	AO% Vas Scores/Pain Bone/Soft Tissue Healing	NR
Zhu J. et al. 2020 China, systematic review and meta-analysis	19 RCTs (811,1343) 6 in Turkey 5 in India 2 in Iran 2 in Cyprus 1 in Egypt 1 in Italy 1 in Lithuania 1 in Brazil	PubMed, Embase, Web of Science, and Cochrane Library Last Search May 2019.	Mean Age 23,66 (No mention in 4 studies) Range 18–40 (No mention in 16 studies) M/f n/m	PRF (Type of flap) Vs Spontaneous healing	Pain, swelling, trismus, Soft tissue healing, AO	NR
Zhu J. et al. 2019 China, systematic review and meta-analysis	19 RCTs (954, 1365) 2 CCTs 5 in Iran 4 in Italy 4 in Turkey 2 in India 1 in Saudi Arabia 1 in Pakistan 1 in New Zealand 1 in Austria 1 in USA 1 in Jordan	PubMed, Embase, Web of Science, and Cochrane Library Last Search November 2018	Mean Age 24,7 (No mention in 6 studies) Range 16–42,8 (No mention in 16 studies) m/f 247/401 (No mention in 4 studies)	Envelope flap Vs Triangular Flap Modified Triangular flap	Operating time Pain (VAS Grade, Number of painkillers) Swelling Trismus AO	Publication bias was not assessed because the included number of observations for each outcome was less than 9.
Canellas J.V.D.S et al., 2020, Brazil, a systematic review and network meta-analysis	37 RCTs (4716, 6175) 6 in Iran 4 in Denmark 4 in Turkey 3 in India 4 in China 3 in Spain 2 in Pakistan 1 in Britain 2 in Norway 1 in Nepal 1 in Lithuania 1 in Sweden 1 in Italy 1 in Peru 1 in Egypt 1 in Japan 1 in Kosovo	EMBASE, Cochrane Library, MEDLINE/PubMed database, Web of Science, Scopus, Latin American and Caribbean Health Sciences Literature database (LILACS), and grey literature. Hand Search Last search 2 Semptember 2019	Mean Age 25,6(No mention in 4 studies) Range 15–65 (No mention in 11 studies) m/f 2136/1826 (No mention in 3 studies)	PRF, DSTB, AMGAN, CHX gel, MChlo gel, PRP, CHX- 0,12 gel, CHX-1 gel, ABS, RBBFGF, COLOIDAL SILVER, ADM, HEAL – ALL, ChlorTetra, PAG, TGE, AMCA, PEPH, PEHB Vs PRF + HA, BC, PRF + CHX, PrRF, Placebo, ORZ gel, Eugenol paste, Iodoform, HY	Alveolar Osteitis	
Del Fabbro M. et al. 2017, Italy, Spain, A Systematic Review and Meta-Analysis	15 RCTs (481,849) 7 CCTs (151,302) 8 in India 5 in Turkey 2 in Italy 2 in Brazil 2 in Iran 1 in Spain 1 in USA 1 in Cyprus	MEDLINE, EMBASE, Scopus, Cochrane Central Register of Controlled Trials, Hand search Last search 8 February 2016	m/f (No mention) Mean age 25,21 (No mention in 8 studies) Range 18–50 (No mention in 3 studies)	PRP, PRF, PRP + bovine HA + mb Vs Spontaneous healing, bovine HA + mb	Soft tissue healing (Index of Landry, Probing depth) Alveolar Osteitis Acute inflammation or infection of the alveolus Pain Hard tissue healing (Percentage of new bone, Indirect measurement of bone metabolism, Bone density)	
Snopek et al., 2021, Denmark, Systematic review and meta-analysis	6 double-blinded RCTs with a split-mouth design (309/618) 1 Turkey 1 Lithuania 1 Brazil 1 Saudi Arabia 2 Iran	PubMed/MEDLINE, Embase, Cochrane Library, Scopus and Web of Science, www.opengrey.eu, www.greylit.org. Manual search in International Journal of Oral and Maxillofacial Surgery, British Journal of Oral and Maxillofacial Surgery, Journal of Oral and Maxillofacial Surgery and Journal of Cranio-Maxillofacial Surgery. Reference lists of included articles was also searched. www.clinicaltrials.gov. The last electronic and manual search was performed on June 3, 2020. No search limitations mentioned.	M/F (129/184) (4 patients who were excluded in a study are not specified by gender) Mean age 23.52 (16–60) outpatients	PRF Vs Spontaneous healing	Adverse effects Frequency of AO, facial swelling, pain (VAS), soft tissue healing, PPD, CAL, GR	
Jakobsen C et al., 2013, Denmark, a systematic review	1 split mouth RCT (7/14) Italy	PubMed without language restrictions from 1 January 2000 to 31 December 2011	6 F/1 M mean age: 30,2 (24–40) outpatients	stem/progenitor cells from the pulps of the patients’ maxillary 3rd molars vs. Spontaneous healing	Infection Cortical bone level Clinical attachment (probing depth) Bone maturity at extraction site after 3 months	.
Morjaria KR. et al. 2014, UK, Systematic Review	2 split mouth RCTs (53/106) 1 USA 1 Brazil	MEDLINE and EMBASE and the Cochrane Central register of controlled trials (CENTRAL) were searched up until August 2011. Randomized controlled trials that included and compared healing post-tooth extraction between a control (no intervention) and a graft and/or membrane (test) were selected. English language, only published trials	1 st study: M/F (5 M/9F) mean age 31y 2nd study: 15–25 years, no data on M/F or mean age outpatients	Bioactive glass Xenograft (Gen-Tech) and resorbable membrane Vs Spontaneous healing	CAL Postoperative pain, infection Bone loss, bone density	The selection of only published trials and those that were in the English language introduces publication bias
Canellas et al. 2017, Brazil, systematic review and meta-analysis	7 RCTs (280p and 485 s) 3 in Turkey 2 in India 1 in Iran 1 in Egypt published from 2010 to 2016	MEDLINE/ PubMed, Cochrane Library, LILACS, ScienceDirect, Current Controlled Trials, Clinical- Trials.gov, EU Clinical Trials Register, CAPES, British Journal of Oral and Maxillofacial Surgery, Journal of Oral and Maxillofacial Surgery, International Journal of Oral and Maxillofacial Surgery, Clinical Oral Investigations, Journal of Dentistry, Journal of Cranio-Maxillofacial Surgery, and Oral Surgery, Oral Medicine, Oral Pathology, Oral Radiology, and Endodontology Last search August 2016 No language restriction	Age range 18–48, m/f 83/132, NM in 2 studies, outpatients, 1 in private dental practice, 6 in University Dental Clinic	PRF Vs Spontaneous healing	pain, alveolar osteitis, facial swelling, bone healing	NR
Domic et al., 2023, Austria, Switzerland, Systematic review and meta-analysis	10 RCTs 1 NRSI (603p and 676 s) 6 in Turkey 1 in Saudi Arabia 1 in India 1 in Italy 1 in Spain 1 in Korea Published from 2014 to 2020	Ovid (MEDLINE and CENTRAL), EMBASE, and Pubmed Last search April 7, 2022 English or German language	Age range 18–71 m/f 247/354 NR in 1 study Outpatients University Dental Clinic	Hyaluronic acid in different application forms (spray, gel) and concentrations (0.2-1%) Vs Spontaneous wound healing, BnzHCl spray, L-PRF, other HyA product	Pain, trismus, edema, inflammatory response, oxidative stress, bleeding time, tissue factor, number of painkillers, wound dehiscence, pus, alveolar osteitis, local lymphadenopathy, mucosa healing score, wound infection, overall discomfort, pain, burning sensation, redness	The certainty of evidence was judged as moderate for pain perception and trismus and as low for the swelling assessment.
Santos Pereira et al., 2023, Brazil, systematic review	1 RCT (10p and 20 s), published at 2019 Saudi Arabia	PubMed, Scopus, Embase, Web of Science, Cochrane Library databases Last search February 28, 2022	Mean age 24 m/f 0/10 outpatients university dental clinic	A-PRF clot Vs Blood clot (spontaneous wound healing)	PPD (1- and 3-month postsurgery), gingival recession (1- and 2-month postsurgery), pain, swelling (7th postoperative day)	
Lu Ye et al., 2024, China, Systematic review and meta-analysis	33RCTs (1430p and 2277 s) 12 in India 7 in Turkey 2 in Brazil 2 in Iran 2 in Cyprus 1 in Italy 1 in Lithuania 1 in Poland 1 in Germany 1 in USA 1 in Sri lanka 1 in Japan 1 in Egypt Published from 2013 to 2022	PubMed, Embase, Cochrane, and Web of Science Last search September 2022 English Language Only RCTs	Age range 16–55 (NR in 6 studies) Mean age 24.59 (NR in 7 studies) m/f 510/697 Setting NR	PRF Vs Spontaneous healing	Pain, swelling, trismus, alveolar osteitis, soft tissue healing, bone healing	The pain, swelling and alveolar osteitis indicators have publication bias (*P* = 0.000, *P* = 0.000, *P* = 0.022)
Chen et al., 2023, China, Systematic review and meta-analysis	5 RCTs (360p and 527 s) 3 in Turkey 2 in China Published 2020 to 2022	PubMed, EMBASE, Cochrane Library, Web of Science, Scopus, National Library of Medicine, OpenGrey, Grey Literature Report last search on 18th July 2023, no language restriction	Age, sex and setting NR	CGF (Concentrated growth factor) Vs Spontaneous healing	Soft tissue healing, bone mineral density, alveolar osteitis, pain, trismus, swelling	NR
Menager et al., 2023, France, Systematic review	10 RCTs and 5 NRSI (612p, 838 s) country NR published from 2011 to 2021	Pubmed and Scopus Studies published in English from January 2010 to January 2022	Mean age 26.6 (NR in 4 studies) Age range 18–49 (NR in 5 studies) setting NR	Triangular flap Vs Envelop flap Flapless	Periodontal status of M2M (PPD, CAL, PI, GI, BoP, gingival recession)	
Yuan et al., 2023, China, Systematic review and meta-analysis	14 RCTs (508p and 1016 s) 5 in Turkey 1 in Egypt 6 in India 1 in Lithuania 1 in Brazil Published from 2015 to 2023	PubMed, EMBASE, Cochrane Library, China National Knowledge Infrastructure and Wanfang databases, reference list of included studies Last search on July 2023	Mean age 24.08 (reported in 10 studies) Age range 18–46 (reported in 6 studies) m/f 302/410 NR in 4 studies	PRF Vs Spontaneous healing	Tissue healing scores on the 7th day, pain, alveolar osteitis	A funnel plot analysis of the PRF compared with the control group for the pain score study resulted in a largely symmetrical funnel plot with no significant publication bias

Abbreviations: RCT, randomized controlled trial; PRF, platelet-rich concentrate; GRADE, Grading of Recommendations Assessment, Development and Evaluation; PD, probing depth; CAL, clinical attachment level; WDPDR, weighted difference pocket depth reduction; NRSI, non-randomized study of intervention; PPD, pocket probing depth; NR, non-reported; PRP, platelet rich plasma; m, male; f, female; ABL, alveolar bone level; GTR, guided tissue regeneration; WDCAL, weighted difference clinical attachment level; WDPD, weighted difference probing depth; M2M, mandibular second molar; e-PTFE, expanded polytetrafluoroethylene; p, patients; s, sites; GBR, guided bone regeneration; PLA, polylactic acid; A-PRF, advanced platelet rich fibrin; L-PRF, leukocyte and platelet rich fibrin; SRP, scaling and root planning; ABD, Alveolar bone defect; BPBM, bovine porous bone mineral; CM, collagen membrane; CCT, controlled clinical trial; PRGF, plasma rich in growth factors; piezo, piezosurgery; HyA, hyaluronic acid; AO, alveolar osteitis; VAS, visual analog scale; BnzHCL, Benzydamine hydrochloride; PI, plaque index; GI, gingival index; BoP, bleeding on probing; DSTB, a drain saturated with Terramycin-Polymyxin; AMGAN, amino acid and sodiumhyaluronate; CHX, chlorhexidine; MChlo gel, gel containing 0.2% chlorhexidine and 10 mg of metronidazole; ABS, Ankaferd Blood Stopper topical agent; RBBFGF, Recombinant bovine basic fibroblast growth factor; ADM, Acellular dermal matrix; ChlorTetra, chlortetracycline ointment; PAG, polylactic acid granule; TGE, topical tetracycline; AMCA, trans-4-amino-methyl-cyclohexane; PEPH, antifibrinolytically active propylic ester of p-hydroxybenzoic acid; PEHB, 3 mg propylic ester of p-hydroxy-benzoic acid; BC, blood clot; ORZ, Ornidazole; mb, resorbable collagen membrane

The operative interventions examined included extraction socket management using GBR or GTR techniques, as well as the application of APCs, intra-socket medicaments, and mesenchymal stem cells derived from dental pulp. None of the reviews assessed the influence of local anesthesia type on clinical outcomes. The control group consisted of cases undergoing spontaneous healing or comparisons between different intervention techniques or placebo.

Surgical access and closure methods focused on various flap designs, with direct comparisons conducted to assess their impact on healing outcomes. None of the reviews assessed the influence of suturing technique on clinical outcomes.

Postoperative maneuvers encompassed scaling and root planning, the administration of systemic antibiotics, and the use of mouth rinses, with these approaches evaluated against cases receiving no additional treatment. None of the reviews assessed the influence of suturing material or timing of removal on clinical outcomes.

### Primary study overlap

The 33 included reviews comprised 422 overlapping index publications, of which 191 were unique. In order to avoid potential double counting of outcomes, we calculated the degree of actual overlap by estimating the CCA of each outcome of interest. For each outcome a citation matrix presenting all the included reviews in columns and index publications in rows was provided. Index publications represented in more than one eligible review were recognized in the citation matrix.

The 6 MAs [26, 36, 44, 47, 54, 57] that investigated the outcome of soft tissue healing comprised 125 overlapping index publications, of which 69 were unique (Table S3). As CCA was estimated at 16.23%, very high overlap was detected. Slight overlap was detected in 2 pairs of reviews, moderate in 1, high in 2 and very high in 10.

The 9 MAs [3, 27, 31, 33–35, 37, 38, 47] that investigated the outcome of PPD comprised 143 overlapping index publications, of which 97 were unique (Table S4). As CCA was estimated at 5.93%, moderate overlap was detected. Slight overlap was detected in 19 pairs of reviews, moderate in 6, high in 2 and very high in 9.

The 6 MAs [3, 27, 33–35, 37] that investigated the outcome of CAL comprised 72 overlapping index publications, of which 49 were unique (Table S5). As CCA was estimated at 9.39%, moderate overlap was detected. Slight overlap was detected in 7 pairs of reviews, moderate in 2, high in 2 and very high in 4.

The 7 MAs [3, 26, 28, 33, 37, 47, 54] that investigated the outcome of ABL comprised 115 overlapping index publications, of which 81 were unique (Table S6). As CCA was estimated at 7%, moderate overlap was detected. Slight overlap was detected in 10 pairs of reviews, moderate in 3, high in 3 and very high in 5.

The 10 MAs [26, 28, 31, 36, 44, 45, 48, 52, 54, 57] that investigated the outcome of pain comprised 160 overlapping index publications, of which 76 were unique (Table S7). As CCA was estimated at 12.28%, high overlap was detected. Slight overlap was detected in 22 pairs of reviews, moderate in 4, high in 4 and very high in 15.

The 9 MAs [26, 28, 29, 31, 36, 44, 45, 52, 54] that investigated the outcome of trismus comprised 146 overlapping index publications, of which 73 were unique (Table S8). As CCA was estimated at 12.50%, high overlap was detected. Slight overlap was detected in 18 pairs of reviews, moderate in 4, high in 2 and very high in 12.

The 12 MAs [26, 28, 29, 31, 43–47, 51, 54, 57] that investigated the outcome of alveolar osteitis comprised 227 overlapping index publications, of which 116 were unique (Table S9). As CCA was estimated at 8.70%, moderate overlap was detected. Slight overlap was detected in 27 pairs of reviews, moderate in 2, high in 8 and very high in 29.

The 8 MAs [26, 28, 31, 36, 44, 45, 52, 54] that investigated the outcome of swelling comprised 140 overlapping index publications, of which 73 were unique (Table S10). As CCA was estimated at 13.11%, high overlap was detected. Slight overlap was detected in 15 pairs of reviews, moderate in 3 and very high in 10.

### Methodological quality assessment

Methodological quality of included studies was judged as high in 9 reviews, moderate in 6 reviews, low in 9 reviews and critical low in 9 reviews (Table 2). Almost all the review authors (32 out of 33) included the components of PICO (Population, Intervention, Comparison, Outcome) into the research questions and inclusion criteria. On the contrary, the funding source was reported only in two reviews, while 17 out of 33 studies did not contain an explicit statement that the review methods were established prior to the conduct of the review.

**Table 2:** Methodological quality assessment with the AMSTAR2 tool

Author (year)	1. PICO components	2. A priori design	3. Rationale for study selection	4. Literature search	5. Duplicate Selection	6. Duplicate Abstraction	7. List of excluded studies	8. Description of included studies	9a. RoB in RCTs	9a. RoB in NRSI	10. Funding sources	11. Appropriate MA methods	12. Used RoB in MA	13. Used RoB in interpreting results	14. Discussion of heterogeneity	15. Publication bias	16. Conflict of Interest	Overall rating
Xiang (2019)	Y	N	Y	N	Y	Y	Y	Y	Y	Includes only RCTs	N	Y	Y	Y	Y	Y	Y	Moderate
Chen (2017)	Y	N	Y	N	N	Y	Y	PY	Y	Includes only RCTs	N	Y	N	Y	N	Y	Y	Low
He (2017)	Y	N	Y	PY	N	Y	Y	PY	Y	Y	N	Y	N	N	Y	Y	N	Low
Al-Hamed (2017)	Y	N	Y	N	N	Y	Y	PY	Y	Includes only RCTs	N	N	Y	Y	N	N	N	Low
Barona-Dorado (2014)	Y	N	Y	PY	N	N	Y	N	PY	Includes only RCTs	N	NM	NM	Y	N	NM	N	Critical Low
Lopes da Silva (2020)	Y	PY	Y	Y	Y	Y	Y	Y	Y	Includes only RCTs	N	Y/RCTs NM/NRSI	Y	Y	Y	N	Y	High
Aloy-Prósper (2010)	Y	N	Y	N	N	N	N	N	N	Includes only RCTs	N	NM	NM	N	N	N	N	Critical Low
Toledano-Serrabona (2021)	Y	N	Y	PY	Y	Y	Y	PY	Y	Includes only RCTs	N	NM	Y	Y	N	N	Y	Low
Lee (2016)	Y	N	Y	N	Y	Y	Y	PY	Y	Includes only RCTs	N	Y/RCTs NM/NRSI	Y	Y	Y	Y	Y	Moderate
Barbato (2016)	Y	PY	Y	Y	Y	Y	Y	PY	Y	Includes only RCTs	Y	Y/RCTs NM/NRSI	Y	Y	Y	N	Y	High
Camps-Font (2016)	Y	PY	Y	Y	Y	Y	Y	PY	Y	Includes only RCTs	N	NM	Y	Y	Y	Y	Y	High
Ramos (2022)	Y	PY	Y	N	Y	Y	Y	PY	Y	Includes only RCTs	N	N/RCTs NM/NRSI	N	N	Y	N	Y	Critical Low
Pang (2022)	Y	PY	Y	PY	Y	N	Y	Y	Y	Y	N	N	N	N	Y	N	Y	Critical Low
Soo-Hoong Low (2021)	Y	PY	Y	PY	Y	Y	Y	Y	Y	PY	N	Y	Y	Y	Y	Y	Y	High
Franchini (2019)	Y	N	Y	PY	Y	Y	PY	Y	Y	Includes only RCTs	N	Y	Y	Y	Y	Y	Y	High
Miron (2017)	Y	N	Y	N	Y	Y	Y	PY	N	N	N	NM	NM	N	Y	NM	Y	Moderate
Danylyuk (2019)	Y	N	N	PY	N	N	Y	PY	N	N	N	NM	NM	N	N	NM	N	Critical Low
Del Fabbro (2011)	Y	N	Y	Y	Y	Y	Y	PY	Y	Y	N	NM	NM	Y	Y	NM	Y	Low
Fujioka-Kobayashi (2021)	Υ	Υ	Υ	Υ	Υ	Υ	Υ	Υ	Υ	Includes only RCTs	N	Y/RCT	Y	Y	Y	Y	Y	High
J. Zhu (2020)	Y	N	Y	N	Y	Y	N	PY	Y	Includes only RCTs	N	Y/RCT	Y	Y	Y	Y	Y	Moderate
Junfei Zhu, (2019)	Y	N	Y	PY	Y	Y	N	PY	Y	Y	N	Y/Y	Y	Y	Y	N	N	Moderate
João Vitor dos Santos Canellas, (2020)	Y	Y	Y	Y	Y	Y	Y	PY	Y	Includes only RCTs	N	Y/RCT	Y	Y	Y	Y	Y	High
Del Fabbro (2017)	Y	N	Y	PY	Y	Y	Y	PY	Y	Y	N	Y/N	Y	Y	Y	Y	Y	High
Snopek (2021)	Y	Y	Y	Y	Y	Y	Y	PY	Y	Includes only RCTs	N	Y/RCT	Y	Y	Y	Y	Y	High
Jakobsen (2013)	Y	N	Y	N	N	N	N	PY	N	N	N	NM/NM	NM	N	N	NM	Y	Critical Low
Morjaria (2012)	Y	PY	Y	PY	Y	Y	Y	PY	N	N	N	NM	NM	Y	N	N	N	Critical Low
J. V. dos S. Canellas (2017)	Y	Y	N	Y	Y	N	Y	Y	Y	Includes only RCTs	N	Y/RCT	N	Y	N	N	Y	Low
Domic (2023)	Y	PY	Y	PY	Y	Y	Y	Y	PY	PY	Y	N	Y	Y	N	N	Y	Moderate
Pereira (2024)	Y	PY	N	PY	Y	Y	N	N	PY	PY	N	NM	NM	Y	N	NM	Y	Critical Low
Lu Ye (2024)	Y	Y	N	PY	Y	Y	N	PY	PY	Includes only RCTs	N	N	N	Y	Y	Y	Y	Low
Liang Chen (2023)	Y	PY	N	Y	Y	N	N	PY	PY	Includes only RCTs	N	N	N	Y	N	N	Y	Low
Menager L. (2023)	Y	PY	N	N	Y	Y	N	N	PY	Includes only RCTs	N	NM	NM	Y	N	N	Y	Critical Low
Yongping Yuan (2024)	N	N	N	PY	Y	Y	Ν	PY	PY	Includes only RCTs	N	N	Y	N	N	Y	Y	Low

AMSTAR 2; I: item; Y: yes; N: no; PY: partial yes. Item 1: did the research questions and inclusion criteria for the review include the components of PICO? Item 2: did the report of the review contain an explicit statement that the review methods were established prior to the conduct of the review and did the report justify any significant deviations from the protocol? Item 3: did the review authors explain their selection of the study designs for inclusion in the review? Item 4: did the review authors use a comprehensive literature search strategy? Item 5: did the review authors perform study selection in duplicate? Item 6: did the review authors perform data extraction in duplicate? Item 7: did the review authors provide a list of excluded studies and justify the exclusions? Item 8: did the review authors describe the included studies in adequate detail? Item 9: did the review authors use a satisfactory technique for assessing the risk of bias (RoB) in individual studies that were included in the review? Item 10: did the review authors report on the sources of funding for the studies included in the review? Item 11: if meta-analysis was performed did the review authors use appropriate methods for statistical combination of results? Item 12: if meta-analysis was performed, did the review authors assess the potential impact of RoB in individual studies on the results of the meta-analysis or other evidence synthesis? Item 13: did the review authors account for RoB in individual studies when interpreting/discussing the results of the review? Item 14: did the review authors provide a satisfactory explanation for, and discussion of, any heterogeneity observed in the results of the review? Item 15: if they performed quantitative synthesis, did the review authors carry out an adequate investigation of publication bias (small study bias) and discuss its likely impact on the results of the review? Item 16: did the review authors report any potential sources of conflicts of interest, including any funding they received for conducting the review?

Risk of bias of primary studies was collected from SRs and presented in Table 3. Cochrane’s Collaboration tool version of 2011 [58] was used in 18 reviews [3, 26–29, 31, 33–36, 38, 39, 44–47, 51, 53] and version 2008 [59] in 1 review [48]. Cochrane’s risk of bias 2 tool (RoB 2) [60] was used in 7 reviews [37, 43, 52, 54–57] and risk of bias in non-randomized of interventions (ROBINS-I) [61]was used in 3 reviews [37, 52, 56]. One review [30] used Jadad Scale [62] and two [42, 50] used a custom tool. Four reviews did not assess risk of bias in included primary studies [32, 40, 41, 49].

**Table 3:** Risk of bias of primary studies collected from systematic reviews

Author	Risk of bias assessment in primary studies (RCTs)	Risk of bias assessment in primary studies (NRSI)	Tool for assessing risk of bias
Xiang et al. 2019	5 with unclear risk of bias 5 with high risk of bias	Included only RCTs	Cochrane Collaboration’s tool (2011)
Chen et al. 2016	1 with low risk of bias 5 with unclear risk of bias 2 with high risk of bias	Included only RCTs	Cochrane Collaboration’s tool (2011)
He et al. 2017	2 with low risk of bias 3 with unclear risk of bias 4 with high risk of bias	1 with high risk of bias	Cochrane Collaboration’s tool (2011)
Al-Hamed et al. 2017	4 with unclear risk of bias 1 with high risk of bias	1 with high risk of bias	Cochrane Collaboration’s tool (2011)
Barona- Dorado et al. 2014	2 with total score 3 1 with total score 4	Included only RCTs	Jadad Scale (1996)
Lopes da Silva et al. 2020	3 with low risk of bias 12 with unclear risk of bias 5 with high risk of bias	Included only RCTs	Cochrane Collaboration’s tool (2011)
Aloy- Prosper et al. 2010	NR		
Toledano-Serrabona et al. 2021	1 with low risk of bias 1 with unclear risk of bias 1 with high risk of bias	Included only RCTs	Cochrane Collaboration’s tool (2011)
Lee et al. 2016	6 with unclear risk of bias 1 with high risk of bias	Included only RCTs	Cochrane Collaboration’s tool (2011)
Barbato et al. 2016	3 with low RoB, 8 with unclear RoB 5 with high RoB	Included only RCTs	Cochrane Collaboration’s tool (2011)
Camps-Font et al. 2018	7 with low RoB, 1 with unclear RoB 13 with high RoB	Included only RCTs	Cochrane Collaboration’s tool (2011)
Ramos et al. 2022	1 with low RoB 13 with unclear RoB 3 with high risk of bias	Included only RCTs	Cochrane Collaboration’s tool (2011)
Pang et al. 2022	7 with low risk of bias 1 with some concerns 1 with high risk of bias	7 with moderate risk of bias 1 with critical risk of bias	Cochrane’s ROBINS-I (2016) for NRSI and RoB 2 (2019) for RCTs.
Soo-Hoong Low et al. 2020	1 with low risk of bias 6 with low risk of bias 1 with high risk of bias	8 with unclear risk of bias 2 with high risk of bias	Cochrane Collaboration’s tool (2011)
Franchini M et al., 2019	3 with high risk of bias	Included only RCTs	Cochrane Collaboration’s tool (2011)
Miron RJ et al. 2016	NR		
Danylyuk Y., 2019	NR		
Del Fabbro M et al. 2011	3 with moderate risk of bias	1 with high risk of bias 2 with moderate risk of bias	Custom risk of bias tool that assessed sample size calculation; concealed allocation of treatment; completeness of information on reasons for withdrawal by trial group; the randomization method (if applicable); the definition of exclusion/inclusion criteria; the comparability of control and treatment groups at entry, and the calibration and blinding of evaluator(s) for outcome assessment. All these criteria were judged as adequate/non adequate.
Fujioka-Kobayashi M et al. 2021	12 with low risk of bias 6 with some concerns	Included only RCTs	Cochrane’s RoB 2 (2019) for RCTs
Zhu J. et al. 2020	13 with low risk of bias 6 with unclear risk of bias	Included only RCTs	Cochrane Collaboration’s tool (2011)
Zhu J. et al. 2019	Overall risk of bias score for each study was not reported		Cochrane Collaboration’s tool (2011)
Canellas J.V.D.S et al., 2020	29 with unclear risk of bias 8 with high risk of bias	Included only RCTs	Cochrane Collaboration’s tool (2011)
Del Fabbro M. et al. 2017	Overall risk of bias score for each study was not reported		Cochrane Collaboration’s tool (2011)
Snopek et al., 2021	6 with unclear risk of bias	Included only RCTs	Cochrane Collaboration’s tool (2008)
Jakobsen C et al., 2013	NR		
Morjaria KR. et al. 2014	2 with high risk of bias	Included only RCTs	Custom tool based on: Cochrane Collaboration’s tool (2008) Jadad Scale (2007) CONSORT statement 2010
Canellas et al. 2017	1 with unclear risk of bias 6 with high risk of bias	Included only RCTs	Cochrane Collaboration’s tool (2011)
Domic et al., 2023	3 with low risk of bias 7 with some concerns	1 with low risk of bias	Cochrane’s ROBINS-I (2016) for NRSI and RoB 2 (2019) for RCTs.
Santos Pereira et al., 2023	1 with high risk of bias	Included only RCTs	Cochrane Collaboration’s tool (2011)
Lu Ye et al., 2024	21 with low risk of bias 9 with some concerns 3 with high risk of bias	Included only RCTs	Cochrane’s RoB 2 (2019) for RCTs
Chen et al., 2023	4 with low risk of bias 1 with high risk of bias	Included only RCTs	Cochrane’s RoB 2 (2019) for RCTs
Menager et al., 2023	1 with low risk of bias 9 with high risk of bias	1 with unclear risk of bias 4 with high risk of bias	Cochrane’s RoB 2 (2019) for RCTs
Yuan et al., 2023	11 with some concerns 3 with high risk of bias	Included only RCTs	Cochrane’s RoB 2 (2019) for RCTs

## Summary of results

The included reviews provided outcome data relating to the application of platelet-rich fibrin (PRF), leukocyte-platelet-rich fibrin (L-PRF), advanced platelet-rich fibrin (A-PRF), platelet-rich plasma (PRP), APC, concentrated growth factors (CGF) and hyaluronic acid (HyA) into the extraction socket and compared them to spontaneous healing or placebo. Additionally, ridge preservation techniques and regenerative procedures were compared to spontaneous healing. Surgical access and closure methods were analyzed by comparing different flap designs, while post-surgical management strategies—including scaling, systemic antibiotic administration, and antiseptic use—were, also, examined. The assessed outcomes included soft tissue healing, change in PPD, change in CAL, alveolar bone healing, postoperative pain, swelling and trismus, as well as the incidence of alveolar osteitis (Tables 4, 5, 6, 7, 8, 9, 10, 11).

**Table 4:** Primary outcome – Pocket Probing Depth

Comparison	Number of sites (primary studies)	Measure of effect	Effect Model	Direction of effect	Follow up	Heterogeneity
Other flap* vs Triangular flap	164 vs 149 (8)	WDPDR= –0.14; 95% CI (–0.44, 0.17)	Random	No difference	At least 3 months	I2 = 83.4%
Triangular vs Envelope flap	49 vs 49 (3)	SMD= –1.36; 95% CI (–2.68, –0.03)	Random	Favors triangular flap (lower PPD)	7 days	I2 = 88%
Triangular vs Envelope flap	37 vs 37 (2)	SMD= –1.43; 95% CI (–3.54, 0.69)	Random	No difference	14 days	I2 = 93%
Ridge preservation** vs Control	173 vs 173 (11)	MD= –1.42; 95% CI (–2.01, –0.83)	Random	Favors ridge preservation	6–72 months	I2 = 97%
APC vs Control	48 vs 49 (2)	MD= 1.72; 95% CI (–0.16, 3.60)	Random	No difference	3–6 months	I2 = 95%

*APC* autologous platelet concentrate, *WDPDR w*eighted mean difference of the probing depth reduction, *SMD* standardized mean difference *MD* mean difference, *PPD* pocket probing depth, *CI* confidence interval

*Szmyd flap, modified Szmyd flap, envelope flap, modified envelope flap, modified triangular flap

**Different techniques and biomaterials were used

**Table 5:** Primary outcome – Clinical Attachment Level

Comparison	Number of sites (primary studies)	Measure of effect	Effect model	Direction of effect	Follow up	Heterogeneity
Other flap* vs Triangular flap	68 vs 53 (3)	WDCAG= 0.05; 95% CI (–0.84, 0.94)	Random	No difference	At least 3 months	I2= 77.4%
Regenerative techniques** vs Control	195 vs 180 (11)	MD= 1.98; 95% CI (1.44, 2.52)	Random	Favors regenerative techniques	4.5–72 months	I2= 87.3%

*WDCAG* weighted mean difference of the clinical attachment gain, *MD* mean difference, *CI* confidence interval

*Szmyd flap, modified Szmyd flap, envelope flap, modified envelope flap, modified triangular flap

**Guided Bone regeneration, guided tissue regeneration, osseous grafting, platelet concentrates

**Table 6:** Secondary outcome – Adverse Events - Pain

Comparison*	Number of sites (primary studies)	Measure of effect	Effect model	Direction of effect	Follow up	Heterogeneity
Envelope vs triangular flap	100 vs 100 (3)	MD= 0.25; 95% CI (–0.82, 1.33)	Random	No difference	1 day	I2=86%
Envelope vs triangular flap	139 vs 139 (5)	MD= –0.01; 95% CI (–0.81, 0.79)	Random	No difference	2 days	I2=80%
Envelope vs triangular flap	140 vs 140 (5)	MD= 0.06; 95% CI (–0.08, 0.21)	Random	No difference	3 days	I2=82%
Envelope vs triangular flap	100 vs 100 (3)	MD= –0.40; 95% CI (–1.20, 0.39)	Random	No difference	4 days	I2=85%
Envelope vs triangular flap	68 vs 68 (2)	MD= –0.28; 95% CI (–0.93, 0.36)	Random	No difference	5 days	I2=52%
Envelope vs triangular flap	68 vs 68 (2)	MD= –0.41; 95% CI (–0.75, –0.07)	Random	Favors envelope flap	6 days	I2=0
Envelope vs triangular flap	181 vs 175 (6)	MD= –0.02; 95% CI (–0.23, 0.19)	Random	No difference	7 days	I2=57%
PRF vs Spontaneous healing	499 vs 499 (15)	SMD= –0.60; 95% CI (–1.00, –0.20)	Random	Favors PRF	1 day	I2=89.1%
PRF vs Spontaneous healing	504 vs 504 (17)	SMD= –0.86; 95% CI (–1.26, –0.46)	Random	Favors PRF	3 days	I2=88.7%
PRF vs Spontaneous healing	473 vs 473 (15)	SMD= –0.76; 95% CI (–1.14, –0.38)	Random	Favors PRF	7 days	I2=86.7%
L-PRF vs Spontaneous healing	67 vs 67 (3)	MD= –0.98; 95% CI (–1.65, –0.32)	Random	Favors L-PRF	1 day	I2=28%
L-PRF vs Spontaneous healing	67 vs 67 (3)	MD= –1.07; 95% CI (–1.53,–0.60)	Fixed	Favors L-PRF	3 days	I2=68%
HyA application vs Control	120 vs 114 (4)	MD= 0.52; 95% CI (–0.34, 1.38)	Random	No difference	2–3 days	I2=0
HyA application vs Control	140 vs 134 (5)	MD= 0.32; 95% CI (0.12, 0.51)	Random	Favors HyA	7 days	I2=0

*VAS* visual analogue scale, *PRF* platelet rich fibrin, *L-PRF* leukocyte - platelet rich fibrin, *MD* mean difference, *SMD* standardized mean difference, *CI* confidence interval, *HyA* hyaluronic acid

*Outcome measure: VAS pain score

**Table 7:** Secondary outcome – Adverse Events - Trismus

Comparison	Number of sites (primary studies)	Measure of effect	Effect model	Direction of effect	Follow up	Heterogeneity
Envelope vs triangular flap*	103 vs 104 (4)	SMD= –0.16; 95% CI (–0.72, 0.41)	Random	No difference	2 days	I2=73%
Envelope vs triangular flap*	65 vs 65 (3)	SMD= –0.12; 95% CI (–0.47, 0.22)	Fixed	No difference	3 days	I2=0
Envelope vs triangular flap*	137 vs 137 (5)	MD= 0.28; 95% CI (–0.41, 0.98)	Random	No difference	7 days	I2=83%
Envelope vs triangular flap**	107 vs 100 (3)	MD= –1.02; 95% CI (–4.25, 2.22)	Random	No difference	7 days	I2= 67%
Envelope vs triangular flap**	39 vs 39 (2)	SMD= 0.25; 95% CI (–0.75, 1.26)	Random	No difference	14 days	I2= 80%
PRF vs Spontaneous healing*	45 vs 45 (2)	SMD= 0.20; 95% CI (–0.56, 0.95)	Random	No difference	7 days	I2=69%
PRF vs Spontaneous Healing*	105 vs 105 (3)	SMD= –0.50; 95% (–0.78, –0.22)	Fixed	Favors PRF	1 day	I2= 17.1%
PRF vs Spontaneous Healing*	60 vs 60 (2)	SMD= –0.34; 95% CI (–0.71, 0.02)	Fixed	No difference	3 days	I2= 0
PRF vs Spontaneous Healing*	155 vs 155 (5)	SMD= –0.26; 95% CI (–0.48, –0.03)	Fixed	Favors PRF	7 days	I2= 18.7%
HyA application vs Spontaneous Healing*	64 vs 64 (3)	MD= 1.31; 95% CI (–0.65, 3.26)	Random	No difference	2–3 days	I2= 25.86%
HyA application vs Spontaneous Healing*	140 vs 134 (5)	MD= 1.08; 95% CI (–0.97, 3.12)	Random	No difference	7 days	I2= 55.99%

*PRF* platelet rich fibrin, *A-PRF* advanced - platelet rich fibrin, *MD* mean difference, *SMD* standardized mean difference, *CI* Confidence Interval, *HyA* hyaluronic acid

*Outcome measure: Postoperative interincisal distance

**Outcome measure: Change in interincisal distance

**Table 8:** Secondary outcome – Adverse Events – Alveolar Osteitis

Comparison	Number of sites (primary studies)	Number of events (primary studies)	Measure of effect	Effect model	Direction of effect	Heterogeneity
Envelope vs triangular flap	313 vs 316 (7)	42 vs 28 (7)	RR= 1.51; 95% CI (0.97, 2.35)	Fixed	No difference	I2=45%
APC vs Control	264 vs 234 (4)	8 vs 33 (4)	RR= 0.20; 95% CI (0.03, 1.18)	Random	No difference	I2= 63%
PRF vs Control	301 vs 301 (6)	25 vs 60 (6)	RR= 0.43; 95% CI (0.28, 0.65)	Fixed	Favors PRF	I2=0

*APC* autologous platelet concentrate, *PRF* platelet rich fibrin, *RR* risk ratio, *CI* confidence interval

**Table 9:** Secondary outcome – Adverse Events – Swelling

Comparison*	Number of sites (primary studies)	Measure of effect	Effect model	Direction of effect	Follow up	Heterogeneity
Envelope vs triangular flap	103 vs 104 (4)	SMD= 0.83; 95% CI (–0.48, 2.14)	Random	No difference	2 days	I2= 94%
Envelope vs triangular flap	68 vs 68 (3)	SMD= 0.35; 95% CI (–0.15, 0.84)	Random	No difference	3 days	I2= 51%
Envelope vs triangular flap	139 vs 140 (6)	SMD= 0.07; 95% CI (–0.23, 0.37)	Random	No difference	7 days	I2= 35%
Envelope vs triangular flap	39 vs 39 (2)	SMD= –0.09; 95% CI (–0.54, 0.35)	Fixed	No difference	14 days	I2= 0
HyA application vs Spontaneous Healing	34 vs 34 (2)	MD= –2.08; 95% CI (–23.73, 19.58)	Random	No difference	2–3 days	I2= 87.94%
HyA application vs Spontaneous Healing	34 vs 34 (2)	MD= 1.75; 95% CI (–14.38, 17.89)	Random	No difference	7 days	I2= 66.86%
PRF vs Spontaneous Healing	412 vs 412 (11)	MD= –1.17; 95% CI (–1.83, –0.51)	Random	Favors PRF	1 day	I2= 91.7%
PRF vs Spontaneous Healing	422 vs 422 (12)	MD= –1.66; 95% CI (–2.43, –0.90)	Random	Favors PRF	3 days	I2=93.2%
PRF vs Spontaneous Healing	422 vs 422 (9)	MD= –1.82; 95% CI (–2.72, –0.92)	Random	Favors PRF	7 days	I2=94.9%

*SMD* standardized mean difference, *HyA* hyaluronic acid, *MD* mean difference, *PRF* platelet rich fibrin, *CI* confidence interval

*Outcome measure: Distance between facial reference points

**Table 10:** Primary outcome – Soft tissue healing

Comparison*	Number of sites (primary studies)	Measure of effect	Effect model	Direction of effect	Follow up	Heterogeneity
PRF vs Spontaneous healing	60 vs 60 (2)	SMD= –0.20; 95% CI (–1.82, 1.43)	Random	No difference	1 day	I2= 94.7%
PRF vs Spontaneous healing	70 vs 70 (3)	SMD= 0.10; 95% CI (–1.38, 1.58)	Random	No difference	3 days	I2=93.6%
PRF vs Spontaneous healing	115 vs 115 (5)	SMD= –0.03; 95% CI (–1.18, 1.13)	Random	No difference	7 days	I2=93.7%
PRF vs Spontaneous healing	90 vs 90 (4)	SMD= 0.08; 95% CI (–1.26, 1.41)	Random	No difference	14 days	I2=93.6%
L-PRF vs Spontaneous healing	50 vs 50 (2)	SMD = –0.70; 95% CI (–3.50, 2.10)	Random	No difference	7 days	I2 =97%
APC vs Spontaneous healing	78 vs 63 (3)	MD= 1.01; 95% CI (0.77, 1.24)	Fixed	Favors APC	7 days	I2=77%

*PRF* platelet rich fibrin, *L-PRF* leukocyte - platelet rich fibrin, *APC* autologous platelet concentrate, *SMD* standardized mean difference, *MD* mean difference, *CI* confidence interval

*Outcome measure: Landry’s healing index, Modified Landry’s healing index

**Table 11:** Primary outcome – Alveolar Bone Level

Comparison	Outcome Measure	Number of sites (primary studies)	Measure of effect	Effect model	Direction of effect	Follow up	Heterogeneity
PRF vs Spontaneous healing	Scintigraphic evaluation of osteoblastic activity	34 vs 34 (2)	WMD = 0.05; 95% CI (− 0.44, 0.55)	Fixed	No difference	28–90 days	I2=0
Regenerative techniques* vs Spontaneous healing	Radiographic evaluation of bone height	188 vs 188 (8)	MD= 1.21; 95% CI (0.21, 2.21)	Random	Favors regenerative techniques	6–12 months	I2=92.7%
APC vs Spontaneous healing	Histomorphometric characteristics of the percentage of new bone	27 vs 11 (2)	MD= 1.55%; 95% CI (–6.37, 9.48)	Fixed	No difference	12 weeks	I2=64%
APC vs Spontaneous healing	Bone density evaluation	50 vs 50 (2)	MD= 5.06; 95% CI (1.45, 8.66)	Fixed	Favors APC	1 month	I2=68%
APC vs Spontaneous healing	Bone density evaluation	50 vs 50 (2)	MD= 6.66; 95% CI (3.11, 10.21)	Fixed	Favors APC	3 months	I2=35%
APC vs Spontaneous healing	Bone density evaluation	50 vs 50 (2)	MD= 7.29; 95% CI (4.31, 10.28)	Fixed	Favors APC	6 months	I2=0
PRF vs Spontaneous healing	Bone density evaluation	120 vs 120 (3)	SMD= 2.34; 95% CI (0.18, 4.51)	Random	Favors PRF	4 months	I2= 97.2%

*APC* autologous platelet concentrate, *PRF* platelet rich fibrin, *MD* mean difference, *SMD* standardized mean difference, *CI* confidence interval

*Guided tissue regeneration, Osseous grafting, Guided bone regeneration

## Surgical access and closure interventions

### Results of systematic reviews with qualitative synthesis

Two reviews included in this overview performed a qualitative analysis assessing the outcomes of surgical access and closure interventions. Aloy-Prosper et al. [32] found that flap design did not influence PPD or CAL on the distal aspect of the M2M following M3M surgery. Menager et al. [56], concluded in conflicting results regarding the effect of flap design on periodontal health status of M2M. Most of the primary studies showed no difference between different flap designs [63–71]. The primary studies that observed significant differences between flaps showed better periodontal measurements in the triangular flap group compared to the envelope flap group [72–75], except for one [76].

### Results of systematic reviews with quantitative synthesis

#### Primary outcome – pocket probing depth

Two reviews examined PPD as an outcome of interest [27, 31]. The comparison between triangular flap and any different flap design showed no statistically significant effect on PPD [WDPDR= −0.14; 95% CI (−0.44, 0.17)] at least 3 months after extraction [27]. No difference was also reported on PPD [SMD= −1.43; 95% CI (−3.54, 0.69)], when triangular flap was compared only with envelope flap 14 days after baseline [31]. On the other hand, one MA of 3 studies (98 extraction sites) comparing the envelope and triangular flap designs revealed significantly lower PPD for the envelope flap group [SMD= −1.36; 95% CI (−2.68, −0.03)] at the 7th postoperative day [31]. (Table 4).

#### Primary outcome – clinical attachment level

One MA including 3 primary studies (121 extraction sites) compared triangular flap with any other flap. No statistically significant difference was found [WDCAG = 0.05; 95% CI (−0.84, 0.94)] after at least 3 months of follow up [27] (Table 5).

#### Secondary outcome – adverse events – pain

The effect of different flap design on pain after third molar surgery was investigated in 2 reviews [31, 45]. All primary studies assessed pain intensity subjectively using a visual analogue scale (VAS). Envelope flap design was compared to triangular flap showing significantly less pain intensity for envelop flap group only on 6th postoperative day [MD= −0.41; 95% CI (−0.75, −0.07)] [31] and no difference on 1 st [MD = 0.25; 95% CI (−0.82, 1.33)] [45], 2nd [MD= −0.01; 95% CI (−0.81, 0.79)] [45], 3rd [MD = 0.06; 95% CI (−0.08, 0.21)] [45], 4th [MD= −0.40; 95% CI (−1.20, 0.39)] [31], 5th [MD= −0.28; 95% CI (−0.93, 0.36)] [31] and 7th [MD= −0.02; 95% CI (−0.23, 0.19)] [45] postoperative days. (Table 6).

#### Secondary outcome – adverse events – trismus

The impact of flap design on trismus following M3M surgical extraction was investigated in 2 reviews [31, 45]. Trismus was measured either as the maximum interincisal distance between the maxillary and mandibular central incisors or as the change in preoperative and postoperative interincisal opening measurements. The comparison between triangular and envelope flap designs revealed no effect on trismus on days 3 [SMD= −0.12; 95% CI (−0.47, 0.22)] [31], 7 [MD = 0.28; 95% CI (−0.41, 0.98); outcome measure was postoperative interincisal distance, MD= −1.02; 95% CI (−4.25, 2.22); outcome measure was change in interincisal distance] [45] and 14 [SMD = 0.25; 95% CI (−0.75, 1.26)] [31] (Table 7).

#### Secondary outcome – adverse events – alveolar osteitis

Flap design effect on incidence of AO was examined in two reviews [31, 45]. Seven primary studies (629 extraction sites) compared the envelope and triangular flap designs and found no difference regarding the incidence of alveolar osteitis [RR = 1.51; 95% CI (0.97, 2.35)] [45]. (Table 8).

#### Secondary outcome – adverse events – swelling

The influence of flap design on postoperative facial swelling was investigated in two systematic reviews [31, 52]. The findings indicated that variations in flap design did not significantly impact post-extraction swelling (Table 9).

## Operative interventions and techniques

### Results of systematic reviews with qualitative synthesis

Nine reviews included in this overview conducted a qualitative analysis to evaluate the outcomes of various extraction socket interventions. Aloy-Prosper et al. [32] recommended the use of regenerative techniques with bone grafts in cases where a pre-existing periodontal defect was present distal to M2M. Del Fabbro et al. [42], highlighted the beneficial effects of platelet concentrates in reducing postoperative pain and discomfort while enhancing hard and soft tissue healing. However, due to substantial heterogeneity in study design, sample size, surgical techniques, and platelet concentrate preparation protocols, they were unable to conduct a meta-analysis (MA). The authors emphasized the need for standardization in experimental design to accurately assess the true regenerative potential of platelet concentrates in extraction sockets.

Morjaria et al. [50] conducted a radiographic analysis of alveolar bone height following the application of synthetic and xenogenic bone grafts in M3M extraction sockets and found no significant difference compared to spontaneous healing. Jacobsen et al. [49], presented limited evidence suggesting faster bone regeneration, increased bone volume, and enhanced maturation of bone in post-extraction sites following the application of mesenchymal stem cells derived from dental pulp. Barona-Dorado et al. [30], noted that the evidence supporting the use PRP for alveolar bone preservation remains inadequate, as the included primary studies demonstrated high risk of bias or failed to show any advantage over spontaneous healing. Miron et al. [40], reported that PRF was superior to PRP in enhancing soft tissue healing and reducing the incidence of AO following M3M surgery. Franchini et al. [39] observed unclear findings regarding the effect of PRP on hard and soft tissue healing and postoperative complications associated with M3M extraction. Danylyuk et al. [41], underscored the positive role of A-PRF and L-PRF in decreasing the incidence of AO, alleviating postoperative pain, and reducing swelling and trismus, while not significantly influencing bone healing or PPD distal to M2M.

Santos Pereira et al. [53], found slight improvements in PPD and gingival recession, along with significantly lower pain and swelling on the seventh postoperative day, though no difference in healing scores between A-PRF-treated sockets and blood clot controls. Chen et al. [55], reported that CGF significantly reduced AO risk, accelerated soft tissue healing, decreased postoperative pain, trismus, and swelling, and increased bone mineral density, compared to spontaneous healing following M3M surgical extraction.

### Results of systematic reviews with quantitative synthesis

#### Primary outcome – soft tissue healing

Soft tissue healing was examined as an outcome of operative interventions and techniques in 6 reviews [26, 36, 44, 47, 54, 57], using Landry’s healing index or its modified Landry’s healing index. The application of PRF to M3M post-extraction sockets did not yield significant improvements in soft tissue healing in day 1 [SMD= −0.20; 95% CI (−1.82, 1.43)] [54], day 3 [SMD = 0.10; 95% CI (−1.38, 1.58)] [54], day 7 [SMD= −0.03; 95% CI (−1.18, 1.13)] [54] and day 14 [SMD = 0.08; 95% CI (−1.26, 1.41)] [54], Similarly, L-PRF application showed no significant effect on soft tissue healing on day 7 [SMD = −0.70; 95% CI (−3.50, 2.10)] [36]. However, one MA that included 3 primary studies and encompassed 141 extraction sites indicated statistically improved soft tissue healing in APC-treated sockets by the seventh postoperative day [MD = 1.01; 95% CI (0.77, 1.24)] [47] (Table 10).

#### Primary outcome – pocket probing depth

Seven reviews investigated PPD as an outcome measure [3, 33–35, 37, 38, 47]. The effectiveness of APC was assessed, but a meta-analysis of two primary studies encompassing 97 extraction sites found no significant impact on PPD after at least three months [MD = 1.72; 95% CI (−0.16, 3.60)] [3]. In contrast, ridge preservation techniques were analyzed in 11 studies involving 346 extraction sites, demonstrating a statistically significant reduction in PPD [MD= −1.42; 95% CI (−2.01, −0.83)] over follow-up periods ranging from 6 to 72 months after extraction [38] (Table 4).

#### Primary outcome – clinical attachment level

Eleven primary studies, covering 375 extraction sites, assessed the impact of regenerative techniques on CAL. The pooled analysis showed significantly lower CAL in regenerative techniques group compared to control group [MD = 1.98; 95% CI (1.44, 2.52)] after 4.5 to 72 months [3]. (Table 5).

#### Primary outcome – alveolar bone level

Alveolar bone healing was assessed in 7 reviews [3, 26, 28, 33, 37, 47, 54], evaluated through radiographic, scintigraphic, and histomorphometric methods. The use of regenerative techniques improved bone height compared to spontaneous healing [MD = 1.21; 95% CI (0.21, 2.21)] 6 to 12 months after extraction [3]. Additionally, the application of APC improved bone mineral density compared to spontaneous healing at 1 [MD = 5.06; 95% CI (1.45, 8.66)] [47], 3 [MD = 6.66; 95% CI (3.11, 10.21)] [47] and 6 months [MD = 7.29; 95% CI (4.31, 10.28)] [47]. Similarly, PRF application showed favorable effects at four months [SMD = 2.34; 95% CI (0.18,4.51)] [54]. On the other hand, based on 2 primary studies (68 extraction sites) the implementation of PRF did not accelerate osteoblastic activity [WMD = 0.05; 95% CI (− 0.44, 0.55)] at 28 and 90 days [26]. No difference was found between APC and spontaneous healing for new bone formation percentages (histomorphometric evaluation) at 12 weeks [MD = 1.55%; 95% CI (−6.37, 9.48)] [47] (Table 11).

#### Secondary outcome – adverse events – pain

The effect of different operative interventions on pain after third molar surgery was investigated in 8 reviews [26, 28, 36, 44, 48, 52, 54, 57]. All primary studies assessed pain intensity subjectively using a visual analogue scale (VAS). PRF application was compared to spontaneous healing, demonstrating significantly lower pain intensity in the PRF group on 1 st [SMD= −0.60; 95% CI (−1.00, −0.20)], 3rd [SMD= −0.86; 95% CI (−1.26, −0.46)] and 7th [SMD= −0.76; 95% CI (−1.14, −0.38)] postoperative days [54]. Similar results were found for L-PRF application compared to spontaneous healing with L-PRF to be associated with less post extraction pain on days 1 [MD= −0.98; 95% CI (−1.65, −0.32)] and 3 [MD= −1.07; 95% CI (−1.53, −0.60)] [36]. The implementation of HyA into the post-extraction socket in combination with different types of carriers was compared to placebo or spontaneous healing. The results were equivocal, as the pooled analysis of 4 primary studies (234 extraction sites) showed no difference on pain intensity on the 2nd and 3rd postoperative days [MD = 0.52; 95% CI (−0.34, 1.38)], whereas the analysis of 5 primary studies (274 extraction sites) showed favorable results for HyA group, which was associated with less pain intensity on 7th postoperative day [MD = 0.32; 95% CI (0.12, 0.51)] [52] (Table 6).

#### Secondary outcome – adverse events – trismus

Trismus as an adverse event of M3M surgical extraction was investigated in 7 reviews [26, 28, 29, 36, 44, 52, 54]. It was measured as either the maximum distance between maxillary and mandibular central incisors of test and control groups after extraction or the change of pre- and post-operative interincisal opening measurement. The comparison of PRF application into the extraction socket to spontaneous healing showed less trismus on days 1 [SMD= −0.50; 95% (−0.78, −0.22)] and 7 [SMD= −0.26; 95% CI (−0.48, −0.03)] for PRF group, but no difference on day 3 [SMD= −0.34; 95% CI (−0.71, 0.02)] [54]. Additionally, no effect on trismus was found from the application of A-PRF on day 7 [SMD = 0.20; 95% CI (−0.56, 0.95)] [36] or the application of HyA on days 2,3 [MD = 1.31; 95% CI (−0.65, 3.26)] and 7 [MD = 1.08; 95% CI (−0.97, 3.12)] [52] after extraction. (Table 7).

#### Secondary outcome – adverse events – alveolar osteitis

Ten reviews investigated alveolar osteitis as an adverse event after M3M surgical extraction [26, 28, 29, 43, 44, 46, 47, 51, 54, 57]. The analysis of four primary studies (498 extraction sites) that compared the application of APC to spontaneous healing found no difference regarding the incidence of alveolar osteitis [RR = 0.20; 95% CI (0.03, 1.18)] [47]. However, a meta-analysis conducted four years later found a statistically significant lower incidence of alveolar osteitis in the PRF application group compared to spontaneous healing [RR = 0.43; 95% CI (0.28, 0.65)] [44] (Table 8).

#### Secondary outcome – adverse events – swelling

The results derived from 6 reviews that investigated the effect of different types of operative interventions on facial swelling showed that PRF application into the extraction socket is able to significantly reduce postoperative swelling on day 1 [MD= −1.17; 95% CI (−1.83, −0.51)], 3 [MD= −1.66; 95% CI (−2.43, −0.90)] and 7 [MD= −1.82; 95% CI (−2.72, −0.92)] [54]. Conversely, HyA application did not significantly affect post-extraction swelling after 2–3 days [MD= −2.08; 95% CI (−23.73, 19.58)] and 7 days [MD = 1.75; 95% CI (−14.38, 17.89)] [52] (Table 9).

## Post-surgical management

### Results of systematic reviews with qualitative synthesis

Two reviews conducted a qualitative analysis of outcomes of post-surgical interventions. Aloy-Prosper et al. [32] found that curettage of the radicular surface of M2M together with oral hygiene instructions improved PPD or CAL on the distal aspect of the M2M following M3M surgery. Pang et al. [37] investigated the effects of postoperative scaling and root planning, antibiotic administration, and chlorhexidine use and found no statistically significant differences in terms of PPD or CAL reduction, nor in the final PPD and CAL measurements after 6 months.

## Reporting biases and certainty of evidence

Publication bias was evaluated across six SRs using Begg’s test [26], Egger’s test [26, 27], Peter’s test [3], or through visual inspection of the funnel plot [3, 27, 28, 54, 57]. The certainty of evidence was assessed using the GRADE tool in one study [26], the Jadad Scale in another [30], and the Oxford Center criteria in a third [34]. Two studies reported the overall quality of evidence; however, they did not employ a formal grading system [35, 52] (Table 1).

## Discussion

This overview summarized the results of 33 SRs and 191 unique primary studies regarding the outcome of different interventions on hard and soft tissue healing and periodontal status of M2M after third molar surgical extraction. APC had a favorable effect on soft tissue and alveolar bone healing [47, 54]. PRF had a positive effect on alveolar bone healing and on all the investigated adverse events, decreasing postoperative pain, trismus, swelling and incidence of alveolar osteitis [54]. Triangular flap design was associated with decreased PPD, while envelope flap with less postoperative pain [31, 45]. All regenerative techniques improved post-extraction PPD, CAL and alveolar bone level [3, 33–35]. Furthermore, HyA application showed a positive effect on postoperative pain [52].

The third molar is the last tooth to erupt in the oral cavity. However, due to various systemic and local factors, it may remain impacted, with prevalence rates ranging from 16.7–68.6% [77, 78]. The surgical extraction of an impacted M3M requires elevation of a full-thickness flap to provide adequate access and visibility for ostectomy, odontotomy, and other surgical maneuvers. However, this process disrupts the vascular supply from the periosteum, compromising blood circulation to the underlying bone, while extraction further eliminates vascularization from the periodontal ligament [79]. These disruptions contribute to postoperative bone resorption and periodontal alterations of the status of M2M. Dental clinicians should be aware of the potential interventions that can mitigate these effects and promote optimal healing.

Among the first and most studied surgical access interventions was flap design. The traditional triangular flap was considered as the gold standard and was compared with envelope flap and any other flap. Quantitative data was provided from three MAs [27, 31, 45]. One analysis showed statistically significant difference between different flap design, regarding PPD on the 7th postoperative day [31]. However, this is of low clinical significance, as this is usually the day of suture removal and the first day that oral hygiene is starting to be applied properly by the patient. Of note, functional stability between the denuded root and soft tissue is achieved approximately 14 days after surgery [80]. Another analysis revealed lower postoperative pain for envelope flap group on the 6th postoperative day [31]. Although envelope flap is more conservative and was expected to be associated with uneventful healing, no significant difference was found comparing flap designs and regarding PPD and CAL at least 3 months after baseline [27], postoperative pain from day 1 to day 7 (except for day 6) after extraction [31], trismus the 2nd, 3rd, 7th and 14th postoperative days [31, 45], swelling the 2nd, 3rd, 7th and 14th postoperative days [31] and the incidence of alveolar osteitis [45]. Another parameter of envelope flap that was not investigated in any study was the possible impact of intrasulcular incision on papilla height mesial to M2M and on CAL buccally to M2M and mandibular first molar, especially in patients with thin gingiva biotype.

With respect to extraction socket management, the application of biomaterials compared to spontaneous healing was effective in reducing the PPD after at least 6 months, improving CAL after at least 4.5 months and bone height after 6 months [3, 38]. MAs pooled results from different types of techniques and biomaterials and could not distinguish which is the best one. Corinaldesi et al. did not find any difference between bioresorbable collagen membrane and non-resorbable e-PTFE membranes [81], while Chun-Teh Lee et al. reported that osseous grafting alone appears to be less effective than GTR [34]. Osseous defect morphology remaining after tooth extraction influence the predictability of periodontal regenerative therapy. Of note, this way surgical extraction procedure is becoming demanding and more expensive, while the possibility of extra complications like exposure of the membrane should be taken into account. Furthermore, Soo-Hoong Low et al., evaluated the age and pre-operative PPD as critical factors in decision making regarding ridge preservation, suggesting that patients with age ≤ 25years-old and preoperative PPD ≥ 7 mm distally to M2M or age ≥ 25 years-old and pre-operative PPD > 5 mm are suitable candidates for ridge preservation after M3M surgery [38].

APCs are becoming more and more popular since their first introduction in dental medicine in 1998 for PRP and 2001 for PRF [82, 83]. The rationale behind their use is to promote natural wound healing, by concentrating and accumulating blood-derived growth factors, cytokines, lysosomes and cells into soft and hard tissues. Only one MA that pooled results from two studies that used PRP and one study that used PRGF found favorable results for APC on soft tissue healing the 7th postoperative day [47]. However, the rest of the analyses found no effect on soft tissue healing the 1 st, 3rd, 7th and 14th postoperative days after baseline [54], neither on PPD 3–6 months after baseline [3]. PRF did not accelerate bone healing, which was evaluated with scintigraphy or histomorphometry, 28–90 days [26] and 12 weeks after extraction [47], respectively. On the other hand, when bone mineral density was evaluated with radiographs, APC implementation showed favorable results in all analyses [3, 47, 54]. Conflicting conclusions regarding the efficacy of APCs may be attributed to the inconsistency in reporting protocol parameters as only for PRF there are twenty-four different production protocols [84]. Thus, the interpretation of the results of individual studies is becoming difficult to be compared.

One MA considered HyA application as an intervention after M3M extraction [52]. Although, increasing attention is put on HyA, due to its anti-inflammatory and antibacterial properties and its positive effects on soft and hard tissue healing, significant difference was found only for pain attenuation the 7th postoperative day. No effect was revealed on pain 2–3 days after extraction, on trismus and on swelling 2–3 and 7 days after extraction. The HyA was applied as a gel or spray or in combination with a carrier like gelatin or collagen sponge or in combination with PRF. The concentration was used ranged from 0.2 to 1% and in all cases primary wound closure was obtained in order to minimize the possibility of the material to wash out. Besides, any potential positive local anti-inflammatory effect did not translate into less swelling or trismus.

Post-surgical care protocols—including enhanced dental hygiene, antibiotic therapy, and chlorhexidine mouthrinses—were anticipated to promote healing adjacent to extraction sites; however, meta-regression analysis at six months revealed no significant improvements in the final probing pocket depth (PPD) [37]. These findings suggest that the adjunctive effects of these interventions may be limited over the long term, potentially being overshadowed by other patient-specific or surgical factors.

Although no meta-analysis has specifically evaluated the effect of suture material and timing of suture removal on the periodontal status of the M2M after M3M extraction, individual studies and narrative reviews provide some insights. Suturing materials can affect wound margin approximation and the level of bacterial colonization at the surgical site, thereby influencing healing outcomes [85]. Studies have demonstrated that while resorbable sutures may reduce bacterial retention compared to non-resorbable options, they can also vary in terms of tensile strength and handling properties, which are critical for maintaining proper wound closure [86]. Furthermore, the timing of suture removal is another key factor. Early suture removal (typically within 7 to 10 days postoperatively) may help reduce the risk of plaque accumulation and subsequent inflammatory reactions around the extraction site, potentially mitigating adverse periodontal changes at the distal aspect of the mandibular second molar. However, premature removal may compromise wound stability, while delayed removal may promote a foreign body reaction that adversely affects periodontal health [87]. A recent randomized controlled trial, including split-mouth designs, suggest a novel suturing technique that promote better wound edge adaptation can reduce postoperative complications such as probing pocket depth increases and attachment loss adjacent to the second molar, emphasizing the need to optimize both the suture material and the removal timing [88]. Further research is warranted to standardize these protocols and determine the optimal balance between suture stability and early removal to maximize periodontal healing while minimizing complications in the mandibular second molar following third molar extraction.

Several limitations should be considered when interpreting our findings. First, our overview incorporated evidence from systematic reviews of RCTs and NRSIs that varied in quality from critically low to high, with the primary studies predominantly exhibiting an unclear to high risk of bias. Additionally, the moderate-to-high overlap of primary studies may introduce reporting biases. Preoperative periodontal status was inconsistently reported across the included studies, as many did not use it as an inclusion criterion in their analyses. Although factors such as age, periodontal condition, oral hygiene, medical history, and local anatomy could influence treatment outcomes, these variables fell outside the primary scope of our review, which focused on interventions applied during third molar surgery rather than on preoperative status.

Moreover, the comparisons presented in Table 4 demonstrated high heterogeneity, likely resulting from variations in study design, intervention protocols, and outcome measurements across the primary studies. A further challenge arose from the use of both MD and SMD to report outcomes. Since MDs are based on uniform measurement scales and SMDs adjust for different scales, direct comparisons between these effect sizes are limited, necessitating cautious interpretation of the pooled estimates. While most authors employed a random-effects model to account for variability, the high heterogeneity and mixed effect size metrics still limit the generalizability of the findings.

Lastly, it is possible that some individual studies may have been missed if they were not captured in the original systematic reviews. Future meta-analyses specifically dedicated to investigating these factors would provide valuable insights for a more comprehensive understanding of periodontal healing after third molar surgery.

## Conclusion

Minimizing trauma to dental and periodontal tissues is essential for any surgical extraction protocol. This overview demonstrated that, among the interventions evaluated, the triangular flap design was associated with reduced PPD, whereas the envelope flap design yielded lower postoperative pain. The application of APCs favorably improved soft tissue and alveolar bone healing and diminished adverse events, while HyA primarily alleviated post-extraction pain. Furthermore, all regenerative techniques assessed were found to enhance PPD, CAL, and alveolar bone level following mandibular third molar surgery. It is important to note, however, that the evidence was heterogeneous, with several reviews being of critically low or low quality, largely based on primary studies with an unclear to high risk of bias. These findings underscore the potential of targeted surgical and regenerative strategies in optimizing periodontal outcomes while mitigating tissue trauma.

## Supplementary Information

Below is the link to the electronic supplementary material.

ESM 1 17.7 KB (docx)

ESM 2 16.9 KB (docx)

ESM 3 40.3 MB (xlsx)

ESM 4 40.3 MB (xlsx)

ESM 5 40.3 MB (xlsx)

ESM 6 1.09 MB (pdf)

ESM 7 40.3 MB (xlsx)

ESM 8 40.3 MB (xlsx)

ESM 9 40.3 MB (xlsx)

ESM 10 40.3 MB (xlsx)

ESM 11 40.3 MB (xlsx)

## Data Availability

No datasets were generated or analysed during the current study.

## Declarations

### PROSPERO registration ID

CRD42022307614.
