# Supplementary material for: Interventions to minimize periodontal defect distal to second molar after mandibular third molar surgery: an overview of systematic reviews
Source: Oral Maxillofac Surg. 2025 Aug 22;29(1):146. doi: 10.1007/s10006-025-01432-5 (PMC12373545; doi:10.1007/s10006-025-01432-5)
Supplement: Supplementary file 1 — 17.7 KB (docx) [file 10006_2025_1432_MOESM1_ESM.docx]

Table S1: Search strategy

1. third molar
2. wisdom tooth
3. mandibular third molar
4. impacted mandibular third molar
5. semi-impacted mandibular third molar
6. ((((third molar) OR (wisdom tooth)) OR (mandibular third molar)) OR (impacted mandibular third molar)) OR (semi-impacted mandibular third molar)
7. tooth extraction
8. exodontia
9. surgical extraction
10. tooth removal
11. (((tooth extraction) OR (exodontia)) OR (surgical extraction)) OR (tooth removal)
12. periodontal defect
13. osseous defect
14. probing depth
15. attachment loss
16. localized periodontitis
17. ((((periodontal defect) OR (osseous defect)) OR (probing depth)) OR (attachment loss)) OR (localized periodontitis)
18. guided bone regeneration
19. guided tissue regeneration
20. bone augmentation
21. periodontal regeneration
22. spontaneous healing
23. local anesthesia
24. flap design
25. incision design
26. suturing technique
27. platelet-rich plasma
28. platelet-rich fibrin
29. platelet-derived growth factor
30. enamel matrix protein derivate
31. emdogain
32. (((((((((((((guided bone regeneration) OR (guided tissue regeneration)) OR (bone augmentation)) OR (periodontal regeneration)) OR (spontaneous healing)) OR (local anesthesia)) OR (flap design)) OR (incision design)) OR (suturing technique)) OR (platelet-rich plasma)) OR (platelet-rich fibrin)) OR (platelet-derived growth factor)) OR (enamel matrix protein derivate)) OR (emdogain)
33. (((((((third molar) OR (wisdom tooth)) OR (mandibular third molar)) OR (impacted mandibular third molar)) OR (semi-impacted mandibular third molar)) AND ((((tooth extraction) OR (exodontia)) OR (surgical extraction)) OR (tooth removal))) AND (((((periodontal defect) OR (osseous defect)) OR (probing depth)) OR (attachment loss)) OR (localized periodontitis))) AND ((((((((((((((guided bone regeneration) OR (guided tissue regeneration)) OR (bone augmentation)) OR (periodontal regeneration)) OR (spontaneous healing)) OR (local anesthesia)) OR (flap design)) OR (incision design)) OR (suturing technique)) OR (platelet-rich plasma)) OR (platelet-rich fibrin)) OR (platelet-derived growth factor)) OR (enamel matrix protein derivate)) OR (emdogain))

| Electronic databases | Search strategy | Limits | Hits |
| --- | --- | --- | --- |
| MEDLINE (via PubMed) | (((((((third molar) OR (wisdom tooth)) OR (mandibular third molar)) OR (impacted mandibular third molar)) OR (semi-impacted mandibular third molar)) AND ((((tooth extraction) OR (exodontia)) OR (surgical extraction)) OR (tooth removal))) AND (((((periodontal defect) OR (osseous defect)) OR (probing depth)) OR (attachment loss)) OR (localized periodontitis))) AND ((((((((((((((guided bone regeneration) OR (guided tissue regeneration)) OR (bone augmentation)) OR (periodontal regeneration)) OR (spontaneous healing)) OR (local anesthesia)) OR (flap design)) OR (incision design)) OR (suturing technique)) OR (platelet-rich plasma)) OR (platelet-rich fibrin)) OR (platelet-derived growth factor)) OR (enamel matrix protein derivate)) OR (emdogain)) | Publication date until 15/08/2024 | 145 |
| ScienceDirect | (“third molar” OR “wisdom tooth”) AND (“tooth extraction” OR “exodontia”) AND (“periodontal defect” OR “probing depth” OR “attachment loss”) AND (“guided bone regeneration” OR “guided tissue regeneration”) | Last search date 15/08/2024 | 83 |
| Scopus | "third molar" OR "wisdom tooth" OR "mandibular third molar" OR "impacted mandibular third molar" OR "semi-impacted mandibular third molar" AND "tooth extraction" OR "exodontia" OR "surgical extraction" OR "tooth removal" AND "periodontal defect" OR "osseous defect" OR "probing depth" OR "attachment loss" OR "localized periodontitis" AND "guided bone regeneration" OR "guided tissue regeneration" OR "bone augmentation" OR "periodontal regeneration" OR "spontaneous healing" OR "local anesthesia" OR "flap design" OR "incision design" OR "suturing technique" OR "platelet-rich plasma" OR "platelet-rich fibrin" OR "platelet-derived growth factor" OR "enamel matrix protein derivate" OR "emdogain" | Last search date 15/08/2024 | 376 |
| Virtual Health Library | (((((((third molar) OR (wisdom tooth)) OR (mandibular third molar)) OR (impacted mandibular third molar)) OR (semi-impacted mandibular third molar)) AND ((((tooth extraction) OR (exodontia)) OR (surgical extraction)) OR (tooth removal))) AND (((((periodontal defect) OR (osseous defect)) OR (probing depth)) OR (attachment loss)) OR (localized periodontitis))) AND ((((((((((((((guided bone regeneration) OR (guided tissue regeneration)) OR (bone augmentation)) OR (periodontal regeneration)) OR (spontaneous healing)) OR (local anesthesia)) OR (flap design)) OR (incision design)) OR (suturing technique)) OR (platelet-rich plasma)) OR (platelet-rich fibrin)) OR (platelet-derived growth factor)) OR (enamel matrix protein derivate)) OR (emdogain)) | Last search date 15/08/2024 | 90 |
| Wiley Online Library | ((((((("third molar") OR ("wisdom tooth")) OR ("mandibular third molar")) OR ("impacted mandibular third molar")) OR ("semi-impacted mandibular third molar")) AND (((("tooth extraction") OR ("exodontia")) OR ("surgical extraction")) OR ("tooth removal"))) AND ((((("periodontal defect") OR ("osseous defect")) OR ("probing depth")) OR ("attachment loss")) OR ("localized periodontitis"))) AND (((((((((((((("guided bone regeneration") OR ("guided tissue regeneration")) OR ("bone augmentation")) OR ("periodontal regeneration")) OR ("spontaneous healing")) OR ("local anesthesia")) OR ("flap design")) OR ("incision design")) OR ("suturing technique")) OR ("platelet-rich plasma")) OR ("platelet-rich fibrin")) OR ("platelet-derived growth factor")) OR ("enamel matrix protein derivate")) OR ("emdogain")) | Last search date 15/08/2024 | 153 |
| Web of Science | ((third molar) OR (wisdom tooth) OR (mandibular third molar) OR (impacted mandibular third molar)) AND ((tooth extraction) OR (exodontia) OR (surgical extraction)) AND ((periodontal defect) OR (probing depth) OR (attachment loss) OR (localized periodontitis)) AND ((guided bone regeneration) OR (guided tissue regeneration) OR (periodontal regeneration) OR (spontaneous healing) OR (local anesthesia) OR (flap design) OR (incision design) OR (suturing technique) OR (platelet-rich fibrin) OR (enamel matrix protein derivate)) | Last search date 15/08/2024 | 78 |
| ProQuest Dissertations and Theses Global | ((((((("third molar") OR ("wisdom tooth")) OR ("mandibular third molar")) OR ("impacted mandibular third molar")) OR ("semi-impacted mandibular third molar")) AND (((("tooth extraction") OR ("exodontia")) OR ("surgical extraction")) OR ("tooth removal"))) AND ((((("periodontal defect") OR ("osseous defect")) OR ("probing depth")) OR ("attachment loss")) OR ("localized periodontitis"))) AND (((((((((((((("guided bone regeneration") OR ("guided tissue regeneration")) OR ("bone augmentation")) OR ("periodontal regeneration")) OR ("spontaneous healing")) OR ("local anesthesia")) OR ("flap design")) OR ("incision design")) OR ("suturing technique")) OR ("platelet-rich plasma")) OR ("platelet-rich fibrin")) OR ("platelet-derived growth factor")) OR ("enamel matrix protein derivate")) OR ("emdogain")) | Publication date until 15/08/2024 | 34 |
| Google Scholar | (“third molar” OR “wisdom tooth”) AND (“tooth extraction” OR “exodontia”) AND (“periodontal defect” OR “probing depth” OR “attachment loss”) AND (“guided bone regeneration” OR “guided tissue regeneration”) | Last search date 15/08/2024 | 407 |
