# Supplementary material for: Interventions to minimize periodontal defect distal to second molar after mandibular third molar surgery: an overview of systematic reviews
Source: Oral Maxillofac Surg. 2025 Aug 22;29(1):146. doi: 10.1007/s10006-025-01432-5 (PMC12373545; doi:10.1007/s10006-025-01432-5)
Supplement: Supplementary file 2 — 16.9 KB (docx) [file 10006_2025_1432_MOESM2_ESM.docx]

**Table S2:** Excluded studies with reasons after full text screening

| **Studies** | **Reason for exclusion** |
| --- | --- |
| Dolan S, Rae E. What are the implications of flap design on post-operative complications when carrying out third molar surgery? Evid Based Dent. 2021 Jan;22(3):104-105. doi: 10.1038/s41432-021-0198-7. PMID: 34561661. | Non-systematic review |
| Piecuch JF. What strategies are helpful in the operative management of third molars? J Oral Maxillofac Surg. 2012 Sep;70(9 Suppl 1):S25-32. doi: 10.1016/j.joms.2012.04.027. PMID: 22916697. | Non-systematic review |
| Hassan KS, Marei HF, Alagl AS. Composite bone graft for treatment of osseous defects after surgical removal of impacted third and second molars: case report and review of the literature. Oral Surg Oral Med Oral Pathol Oral Radiol Endod. 2011 Dec;112(6):e8-15. doi: 10.1016/j.tripleo.2011.04.010. Epub 2011 Jul 23. PMID: 21784674. | Non-systematic review |
| Moraschini V, Barboza ES. Effect of autologous platelet concentrates for alveolar socket preservation: a systematic review. Int J Oral Maxillofac Surg. 2015 May;44(5):632-41. doi: 10.1016/j.ijom.2014.12.010. Epub 2015 Jan 24. PMID: 25631334. | Oral surgery procedures other than third molar surgery |
| Dragonas P, Katsaros T, Avila-Ortiz G, Chambrone L, Schiavo JH, Palaiologou A. Effects of leukocyte-platelet-rich fibrin (L-PRF) in different intraoral bone grafting procedures: a systematic review. Int J Oral Maxillofac Surg. 2019 Feb;48(2):250-262. doi: 10.1016/j.ijom.2018.06.003. Epub 2018 Jul 7. PMID: 30058532. | Oral surgery procedures other than third molar surgery |
| Dragonas P, Schiavo JH, Avila-Ortiz G, Palaiologou A, Katsaros T. Plasma rich in growth factors (PRGF) in intraoral bone grafting procedures: A systematic review. J Craniomaxillofac Surg. 2019 Mar;47(3):443-453. doi: 10.1016/j.jcms.2019.01.012. Epub 2019 Jan 17. PMID: 30711470. | Oral surgery procedures other than third molar surgery |
| Theodosaki AM, Tzemi M, Galanis N, Bakopoulou A, Kotsiomiti E, Aggelidou E, Kritis A. Bone Regeneration with Mesenchymal Stem Cells in Scaffolds: Systematic Review of Human Clinical Trials. Stem Cell Rev Rep. 2024 May;20(4):938-966. doi: 10.1007/s12015-024-10696-5. Epub 2024 Feb 26. PMID: 38407793; PMCID: PMC11087324. | Oral surgery procedures other than third molar surgery |
| Shah N, Cairns M. Autologous Platelet Concentrates to improve post extraction outcomes. Evid Based Dent. 2018 Dec;19(4):118-119. doi: 10.1038/sj.ebd.6401347. PMID: 30573863. | Non-systematic review |
| Hur Y, Ogata Y. Different flap designs have no impact on periodontal outcomes on second molars after impacted third-molar extraction. J Am Dent Assoc. 2017 Nov;148(11):849-852. doi: 10.1016/j.adaj.2017.07.024. PMID: 29080607. | Non-systematic review |
| Santinoni CD, Oliveira HF, Batista VE, Lemos CA, Verri FR. Influence of low-level laser therapy on the healing of human bone maxillofacial defects: A systematic review. J Photochem Photobiol B. 2017 Apr;169:83-89. doi: 10.1016/j.jphotobiol.2017.03.004. Epub 2017 Mar 7. PMID: 28292696. | Oral surgery procedures other than third molar surgery |
| Kalantar Motamedi MR, Heidarpour M, Siadat S, Kalantar Motamedi A, Bahreman AA. Orthodontic Extraction of High-Risk Impacted Mandibular Third Molars in Close Proximity to the Mandibular Canal: A Systematic Review. J Oral Maxillofac Surg. 2015 Sep;73(9):1672-85. doi: 10.1016/j.joms.2015.03.031. Epub 2015 Mar 24. PMID: 25882437. | Third molar extraction was not surgical |
| Blanchy, T., Babilotte, J., Fénelon, M., Marteau, J. M., Fricain, J. C., & Catros, S. (2016). Intérêt des éponges de collagène pour prévenir la résorption osseuse alvéolaire post-extractionnelle: revue systématique de la littérature. *Médecine Buccale Chirurgie Buccale*, *22*(3), 221-232. | Systematic review of animal studies |
| Richardson DT, Dodson TB. Risk of periodontal defects after third molar surgery: An exercise in evidence-based clinical decision-making. Oral Surg Oral Med Oral Pathol Oral Radiol Endod. 2005 Aug;100(2):133-7. doi: 10.1016/j.tripleo.2005.02.063. PMID: 16037768. | Third molar extraction was assessed as intervention |
| Razali, N. S., Younis, L. T., & Ariffin, M. H. Z. (2024). The effectiveness of hydroxyapatite in alveolar ridge preservation: A systematic review. *Journal of International Oral Health*, *16*(1), 19-32. | Oral surgery procedures other than third molar surgery |
| Almontashri, S. M., Aldossary, N. M., & Assyria, A. A. (2023). Comparing the outcomes of Conventional Coronectomy and Graft Coronectomy: a systematic review. *The Open Dentistry Journal*, *17*(1). | Oral surgery procedures other than third molar surgery |
